# Virtual group–based mindfulness for autistic adults: A randomized controlled trial

**DOI:** 10.1177/13623613251340101

**Published:** 2025-07-01

**Authors:** Yona Lunsky, Madelaine Carter, Sue Hutton, Jan Wozniak, Sheng Chen, Jonathan Weiss

**Affiliations:** 1 Centre for Addiction and Mental Health, Canada; 2 University of Toronto, Canada; 3 York University, Canada

**Keywords:** autism, mental health, mindfulness, virtual care

## Abstract

There is evidence for mindfulness as a mental health intervention for autistic adults, but most of the research has focused on in-person interventions. This randomized controlled trial evaluated the impact of a 6-week manualized autistic-informed group-based virtual mindfulness intervention in comparison to a waitlist control. Sixty-three autistic adults from across Canada were randomly assigned to treatment or waitlist. Both groups completed surveys prior to randomization, at 7 and 15 weeks, and the waitlist group was followed for two additional time points after receiving mindfulness. We compared outcomes at the first three time points by group using linear mixed models with a secondary analysis including outcome measures from the waitlist group after intervention. Improvements in overall distress and stress, as well as self-compassion, mindfulness, and mental wellbeing were reported in the treatment condition, maintained at follow-up. No changes were reported in the waitlist condition. Neither group reported changes in terms of autistic commnity connectedness, or interoceptive sensitivity following intervention or follow-up. This study supports the virtual delivery of autistic-informed mindfulness-based programs. Further work could explore the unique benefits of synchronous group–based virtual mindfulness in contrast to more asynchronous ways to build mindfulness skills and in-person instruction.

**Lay abstract:**

Some studies have shown that learning mindfulness helps autistic people, but most of these studies were done in person. We wanted to know if learning mindfulness online in a group would help improve autistic adults’ mental health. We randomly put people into two groups (a waitlist and people who got to be in the mindfulness group right away). This means people did not get to pick which group they went into. A total of 63 autistic adults were in the project. After 6 weeks of mindfulness, the people who learned mindfulness said they felt less stressed, and they were more mindful, and had better wellbeing, and more compassion or kindness for themselves, and these changes were still there 8 weeks later. The people who did not get to do mindfulness right away did not say they felt any different. Neither group said they felt more connected to other autistic people and neither group said they were more aware of the sensations they could feel inside their bodies. This tells us that we can teach groups of autistic people to do mindfulness online together, and it can help at least some autistic adults with their mental health. We need to do more research to find out how much mindfulness training people need, if it is better to learn in person or online, and if it is better to learn as part of a group or alone.

It is well recognized that autistic individuals experience high rates of mental health issues (Curnow et al., 2023; Lai et al., 2019), but that needed mental health services can be inaccessible to them (Dückert et al., 2023). Most of the research on autism and mental health has focused on the mental health of caregivers and of children, with much less research focused on autistic adults. The topic of mental health is a priority for autistic people, and there have been several reviews focused on how to adapt mental health care including psychological therapies for this group (Benevides et al., 2020; Brede et al., 2022). Research with autistic people on their therapy experiences has highlighted the importance of being valued and understood by the therapist, which included them having a good understanding of autism, recognition that talking in session can be difficult, and the importance of session format, including clear session structure (Mazurek et al., 2023). In that same study, mindfulness-based activities were considered helpful by some individuals because of the concrete breathing activities leading to physiological relaxation, in addition to the emphasis on the present moment (Mazurek et al., 2023). Other reviews of mental health interventions have also highlighted the potential value of mindfulness for autistic people (Benevides et al., 2020; Hartley et al., 2019).

Following some early research conducted in the Netherlands on mindfulness-based therapies adapted for autistic adults (Kiep et al., 2015; Spek et al., 2013), a number of studies have been conducted in Canada (Lunsky et al., 2022), the United Kingdom (Gaigg et al., 2020), the United States (Beck et al., 2020; Braden et al., 2022; Conner & White, 2018), the Netherlands (Sizoo & Kuiper, 2017), Sweden (Agius et al., 2023), and Australia (Edwards et al., 2024). These studies have varied in terms of their degree of intervention adaptations for autistic people, whether they were carried out individually or in group, and whether they were in person or online.

Recognizing that in-person mental health interventions can be effective for autistic people, there are also benefits to participating in virtually based interventions, which have become even more evident during the pandemic (Ali et al., 2023; Harris et al., 2022). Virtual groups have the added benefit of not requiring travel for people who have difficulties with travel because of anxiety, accessibility, geographical distance, or financial constraints. For some autistic individuals, virtual engagement can be preferred over in-person interactions because of the flexibility that virtual interactions allow. Asynchronous interactions mean that when a person engages in therapy, it is up to them to decide how and when to participate, with there being no requirements to speak or be on camera. Information can be processed at a pace comfortable for the individual, and material can be reviewed as much as needed. One study of asynchronous mindfulness found early evidence of its feasibility and efficacy (Gaigg et al., 2020). Another study on asynchronous self-compassion, adapted for autistic people and which included weekly check-ins, found that it was helpful, with high rates of acceptance among participants (Cai et al., 2024). Synchronous virtual interactions, although they occur at a set time each week, can also offer flexibility in terms of how someone communicates with the option to have the camera on or off, the option to communicate through typing instead of speaking, and the ease of being able to participate in a comfortable space, without the need to travel. Some psychological interventions, individual and group-based, are now offered virtually in a synchronous manner and are, in fact, preferred by some autistic individuals (Stecher et al., 2023; Westerberg et al., 2023).

As part of our ongoing research on mindfulness and autism, we co-designed an autistic-informed synchronous group intervention which was virtual, and co-delivered by a non-autistic mindfulness therapist together with an autistic advisor. We demonstrated that it was feasible to deliver the intervention, and that the virtual format was acceptable to participants. Participants reported improvements in mindfulness, self-compassion, and psychological distress, with changes maintained at 3 months follow-up. However, this prior study lacked a control group, so it is not known whether the positive impacts reported were specific to the mindfulness-based intervention itself or to other factors, including the passage of time or being part of a supportive group of autistic peers.

Building off of our earlier work, we conducted a randomized controlled trial (RCT) of this virtual autistic-informed mindfulness-based group intervention, in comparison to a waitlist (WL) control. Whereas our earlier research was conducted prior to and during the first year of the pandemic, this work was carried out during pandemic recovery, after many individuals had experienced interacting in and receiving mental health care in virtual spaces. We maintained the outcome measures from the 2022 study (psychological distress, self-compassion, mindfulness), keeping overall psychological distress as the primary outcome.

## Additional measures

Based on lessons learned from our prior mindfulness work and informed by autistic advisors and relevant mindfulness research, we added three additional measures to this study. Autistic community connectedness refers to one’s sense of belonging, social connectedness and political connectedness with other autistic people and the autistic community (Botha et al., 2022). Research demonstrates that autistic connectedness is associated with greater mental wellbeing in autistic adults (Cage et al., 2022). Given the qualitative feedback in the prior study that autistic people appreciated the sense of community that they felt with the other autistic people in the mindfulness program, autistic community connectedness was included in the current study as a secondary outcome. Autistic adults have prioritized mental wellbeing as an important clinical outcome to be included in mental health intervention research (Pellicano & Heyworth, 2023). Although not in the majority of autistic mindfulness studies, a recent self-compassion–based group intervention for autistic people showed improvements in mental wellbeing (Cai et al., 2024). We therefore added a brief measure of mental wellbeing to the current study, which has also been included in other studies with autistic adults (Cage et al., 2022; Maitland et al., 2021). The final secondary outcome added to this study was interoception, the ability to be aware of internal sensations in the body and respond to them. Some autistic people can struggle with interoception, making it challenging for them to self-regulate, which can impact their mental health (Bonete et al., 2023; Garfinkel et al., 2016). Although mindfulness training has led to changes in interoception in non-autistic individuals (Bornemann et al., 2015), and a recent RCT focused on aligning people’s interoceptive experience reported reduced anxiety in autistic adults (Quadt et al., 2021), no studies to date have explored whether mindfulness training improves interoception in autistic people.

Given the building evidence for mindfulness interventions (MI) for autistic adults with only a limited number of studies including a control condition, we hypothesized that participants in the mindfulness group would report significantly greater improvement following intervention that would be maintained at follow-up 8 weeks later, compared to the waitlist (WL) control group. Since the WL control group also received the MI after the 8-week follow-up time point, we also explored the impact of the intervention on their clinical outcomes in a secondary analysis. We hypothesized that they would report similar improvements.

## Methods

This study was a single-center superiority randomized controlled trial with a treatment and WL group, registered at https://clinicaltrials.gov/study/NCT05490615. The goal was to recruit 80 individuals. The proposed sample size was determined based on pre- and post-stress scores from the feasibility study (effect size = 0.72) and a medium effect size (Cohen’s *d* = 0.65) (*N* = 78).

### Participants

To be eligible for the study, participants needed to (1) have a diagnosis of autism or self-identify as autistic, (2) have no intellectual disability, (3) be age 18 years or above, (4) live in Canada, (5) be able to complete online measures in English independently or with telephone or video support from the research coordinator, and (6) be able to participate in a synchronous online intervention with Internet access and a computer, tablet, or smartphone. We did not include individuals who had participated in our feasibility study in the current project.

### Intervention description

As described in Lunsky et al. (2022), the mindfulness program, based on the Mindfulness-Based Stress Reduction (MBSR) curriculum (Kabat-Zinn, 2013), was modified to make it more accessible for autistic adults. There were fewer sessions (six instead of eight), each session was 60 min instead of 120 or 150 min, there was no silent retreat, and all guided meditations practiced in session and recorded practices for homework were shorter. There were always different options presented on how to do each exercise, so if something felt uncomfortable, it could be tried in a different way. Because the group was virtual, and to be supportive of different forms of communication, facilitated discussion could be done by speaking or typing.

Each session began with a group check-in, a brief mindfulness activity (review from the week prior), and a homework discussion. The participant workbook included homework tracking, but participants did not submit documentation of homework completion as part of the study. Next, a new theme was introduced for that week, with a longer formal practice and inquiry discussion. Sessions ended with a summary of the session and an outline of the week’s homework. Homework included both formal and informal practices. The workbook included links to audio recordings of 11 brief formal practices ranging from 2 to 12 min in length available on multiple music platforms and quotes from autistic people about the exercises. With regard to fidelity, all sessions were led by the same mindfulness instructor (S.H.) and autistic advisor (J.Wo.) and followed the outline in the participant workbook but were not formally assessed. Table 1 shows a brief outline of the sessions.

**Table 1.** Weekly outline of mindfulness group sessions.

Week	Weekly topic	Weekly content
1	How mindfulness can help me: Using my senses to calm myself	Introductions—“What does calm mean to me?” Introduction to mindfulness Instruction of formal meditations: • Eating meditation (with option to alternatively use senses like vision and touch) • Ocean breath meditation—use of sound and sensations of breath Inquiry and discussion
2	Breathing to calm myself	Review of previous week’s formal practices Instruction of formal meditations: Introduction to breath practices using various ways to concentrate on the breath • Lotus breath (kinesthetic movement) • Figure 8 breath (movement synched with eyes following the movement) • Hoberman sphere breath (visual anchor) Inquiry and discussion
3	Being aware of my body	Review of previous week’s formal practices Instruction of formal meditations: • Tense & Release (progressive muscle relaxation) with melting ice cream imagery or sensing body directly • Body scan Inquiry and discussion
4	Mindfully moving my body and watching my thoughts	Review of previous week’s formal practices Instruction of formal meditations: • Movement meditation. Introduction of walking/(rolling if mobility device like a wheelchair used) with different sense anchors: -Sensing soles of the feet -Sensing top of the head -Counting and breathing with steps -Use of a phrase/word while stepping Inquiry and discussion
5	Being kind to myself and others	Review of previous week’s formal practices Instruction of formal meditation: • Kindness meditation. Introduction of a meditation technique focused on kindness through training to observe sensations in body related to: -thoughts of kindness toward a person/self -Picturing a person who invokes kindness -Use of a phrase fostering kindness. Inquiry and discussion
6	Mindfulness, music, and art	Review of previous week’s formal practices Instruction of formal meditations: • Drawing the breath Introduction of an exercise synchronizing awareness of the breath with movement of a pen on paper with eyes closed. Inquiry and discussion

### Procedure

The study was advertised and shared through agencies across Canada that work with autistic people and their families. Flyers advertising the groups were also shared through social media (e.g. Twitter and Facebook). Interested participants either completed a REDCap Expression of interest form or contacted the research coordinator to determine eligibility via email, text, or phone. Participants who met eligibility criteria were sent a consent form via email which was then reviewed with the coordinator and signed virtually. Once the consent form was signed and returned to the coordinator, they were sent a survey link and a unique ID to complete baseline measures the week prior to the first session. They were randomized following baseline survey completion to the MI or the WL. Post-measures were completed online in the week after the last session (Week 7; Time 2), and follow-up measures were completed 8 weeks after the final session (Week 15; Time 3). Participants in the WL condition also completed measures after completing mindfulness (Week 22; Time 4) and 8 weeks later (Week 30; Time 5). Online questionnaires were administered through REDCap. Participants were enrolled in three waves (October 2022 to November 2022; November 2022 to January 2023; and January 2023 to February 2023). These time periods were due to practical restraints related to the timing of groups not overlapping with holidays and the need to complete delivery of all of the mindfulness groups by spring of 2023. A maximum of 30 people could be consented at each time point to ensure that the size of the mindfulness groups would not be too large. At the time of providing consent, participants within each cohort knew about the mindfulness group start dates and times for both the treatment and WL conditions. They were allocated, after completing baseline data, to one of the two arms (1:1) via computer-generated stratified randomization through REDCap™. Stratified randomization was used to achieve balance between genders, stratifying women and all other genders (1:1). This was decided as there were a larger number of women interested in study compared to men and gender-diverse individuals. The allocation sequence, enrollment, and assignment were carried out by the research coordinator, who was not involved in the delivery of the intervention.

Baseline, post, and follow-up data collection concluded in June 2023 for those in the mindfulness arm. Data collection concluded for the final waitlisted group in August 2023, after they completed the mindfulness program, and final follow-up measures. Participants received $20 for measure completion at each time point.

### Measures

#### Primary outcome

##### 21-Item Depression, Anxiety & Stress Scale

The 21-item Depression, Anxiety & Stress Scale (DASS-21; Henry & Crawford, 2005), which included seven depression, seven anxiety, and seven stress items, was used to measure overall psychological distress at each time point. Items were rated using a four-point Likert-type scale ranging from 0 (“Did not apply to me at all”) to 3 (“Applied to me very much, or most of the time”) and were summed to yield a single total score between 0 and 63, with higher scores indicating greater levels of distress. Internal consistency of the 21-item DASS was excellent for the current sample at baseline (Cronbach’s α = 0.916). Subscale scores were also computed for depression, anxiety, and stress separately.

#### Secondary outcomes

##### 24-Item Five-Facet Mindfulness Questionnaire-Short Form

The 24-item Five-Facet Mindfulness Questionnaire-Short Form (FFMQ-SF; Baer et al., 2006) was used to assess five components of mindfulness: observing one’s experiences, describing one’s experiences, acting with awareness, accepting inner experiences in a nonjudgmental way, and accepting inner experiences without reacting. Participants were asked to rate how generally true each item was for them using a five-point scale ranging from 1 (“Never or very rarely true”) to 5 (“Very often or always true”), with higher total scores suggesting greater mindfulness (total score range from 24 to 120). Internal consistency of the FFMQ-SF was good for the current sample at baseline (Cronbach’s α = 0.823).

##### Self-Compassion Scale–Short Form

The Self-Compassion Scale–Short Form (SCS-SF; Raes et al., 2011) is a 12-item measure that assesses the ability to demonstrate care and kindness toward oneself, and acceptance of one’s own imperfections. Self-compassion is often a focus of mindfulness-based interventions because of the interrelatedness with state mindfulness (Neff, 2003). Responses were given using a five-point Likert-type scale that ranged from 1 (“Almost never”) to 5 (“Almost always”), yielding a total score between 12 and 60, with higher scores indicating greater self-compassion. Internal consistency of the SCS-SF was good for the current sample at baseline (Cronbach’s α = 0.857).

##### Short Warwick–Edinburgh Mental Wellbeing Scale

The Short Warwick–Edinburgh Mental Wellbeing Scale (SWEMWS) (Stewart-Brown et al., 2009) is a shorter version (seven items) of the 14-item scale, and it assesses participants’ positive mental health. Respondents rate the amount of time in the past 2 weeks that they have experienced a certain thought or feeling on a 5-point Likert-type scale that ranges from 1 (“None of the time”) to 5 (“All of the time”), yielding a total score between 7 and 35, with higher scores indicating greater mental wellbeing. This scale is sensitive to change in psychological interventions and has been used in other studies with autistic adults (Maitland et al., 2021). Internal consistency of the SWEMWS was good for the current sample at baseline (Cronbach’s α = 0.841).

##### Autistic Community Connectedness

Autistic Community Connectedness (ACC) is a 10-item measure developed by autistic researchers to capture how connected study participants felt to the autistic community (Botha, 2020). Each item is rated on a 6-point Likert-type scale from 1 “strongly disagree” to 6 “strongly agree.” Higher scores reflect greater connectedness and are associated with higher mental wellbeing. Internal consistency at baseline in the current study was very good (Cronbach’s α = 0.916).

##### Interoception Sensory Questionnaire-8

The Interoception Sensory Questionnaire (ISQ-8; Suzman et al., 2021) is a brief single-dimension 8-item measure of interoception, based on the original 20-item interoception sensory questionnaire (Fiene et al., 2018) which captures the extent to which autistic adults experience confusion about their interoceptive bodily states. Items explore differences in interoceptive processing using a 7-point Likert-type scale (1 = “Not true at all of me” and 7 = “Very true of me.”) with three reverse-scored items. Higher scores indicate more difficulty registering or interpreting interoceptive sensations. Internal consistency at baseline in the current study was very good (Cronbach’s α = 0.904).

### Community involvement

Autistic individuals were involved in this study. The original mindfulness-based intervention was co-designed with two autistic individuals (Lunsky et al., 2022), and the current trial was co-delivered with an autistic advisor/coauthor, similar to prior iterations. Outcome measures used in the study were informed by prior research and program feedback with autistic people, in addition to input from the autistic coauthor for the current study. This individual worked closely with the other research team members to finalize the outcome measures used in the study, interpret study results, and develop this article. Study recruitment materials and consent forms were reviewed by autistic advisors who work at the hospital prior to submission to the research ethics board.

### Data analysis

This was an intent-to-treat analysis which included all randomized subjects (including those with missing values). Linear mixed models were used to analyze the effects of the interaction between group and time, with group as the between subject factor (MI vs. WL) and time as the within subject factor (Baseline, Week 7 (post-intervention), and Week 15 (follow-up)). Partial Eta-squared effect sizes were calculated with 0.01 considered to be a small effect, 0.06 a medium effect, and 0.14 a large effect size (Cohen, 1992). Simple comparisons were also calculated post hoc for any significant interactions, with the Bonferroni correction.

The primary analysis, which has the most statistical rigor, included the data from the two parallel groups (MI and WL) and three time points (Baseline, Week 7 and Week 15), for all primary and secondary outcomes. For ethical reasons, the WL group completed the program after the treatment group completed the trial. Hence, in a secondary analysis, the data of treating the WL were pooled with the treatment group (MI) at Week 7 and Week 15 and fitted to a linear mixed model. It is noted that the treatment effect in the secondary analysis might be confounded by the time effect in the WL group.

## Results

### Baseline demographics

We were able to recruit participants through social media and our newsletter in the allotted time frame (between October 2022 and February 2023). Of the 95 individuals who expressed an interest in the study by completing the eligibility survey, calling, or emailing the research team, 85 met eligibility criteria and 67 provided informed consent (Cohort 1 = 23, Cohort 2 = 28, Cohort 3 = 16). Of those, 63 completed baseline measures and 34 were randomized to MI and 29 were randomized to WL. Autistic adults in the treatment group participated in the mindfulness program approximately 1 week following randomization. There were no significant differences in demographic characteristics of participants at baseline (Table 2).

**Table 2.** Participant demographic variables.

Characteristics	MI (*n* *=* 34)	WL (*n* *=* 29)	Total (*N* = 63)	*t* or χ^2^	*p*
Age (M, SD) Range	37.32 (11.45) 22–68	37.52 (11.18) 19–61	37.41 (11.26) 19–68		
Gender identity				0.81	0.67
Men	6	7	13		
Women	18	16	34		
Non-binary/gender non-conforming	10	6	16		
Housing status				3.20	0.20
Live alone	10	4	14		
Live with partner/friends	13	10	23		
Live with family	11	15	26		
Marital status				2.38	0.31
Single	21	13	34		
Married	10	14	24		
Divorced	2	1	3		
Separated	1	0	1		
Widowed	0	1	1		

Just over half of participants (54%) identified as women, 25% as gender diverse and 31% as men, ranging in age from 19 to 68 years. Over half of the sample had never been married and 41% were living with family at the time of the study. Seventy-one percent of participants listed at least one co-occurring psychiatric diagnosis, with the most common diagnoses being mood and anxiety-related. The majority of participants (87%) reported at least some prior mindfulness experience, with yoga and using a mindfulness app being most common.

### Treatment adherence

Adherence was assessed in terms of attendance and program completion. Attendance at four or more of six sessions was considered completion. One individual dropped out of the mindfulness program before it started because of life circumstances and one withdrew prior to the first session because they were no longer interested in mindfulness. One individual discontinued because they did not like it, and another discontinued because it was not in French. Two other individuals completed the program but did not complete surveys, as shown in the CONSORT diagram (Figure 1). In total, 31 of the 34 individuals (91.2%) assigned to the treatment arm attended at least four sessions, although three of them did not complete surveys after intervention or at follow-up. Differences in dropout rates between the MI group (6/34) compared to the WL group (3/29) were not statistically significant (χ^2^ = 0.86, p = 0.41) post-randomization. In terms of the secondary analysis, 24 of the 29 individuals assigned to the WL condition completed the program (82.7%).

**Figure 1.**
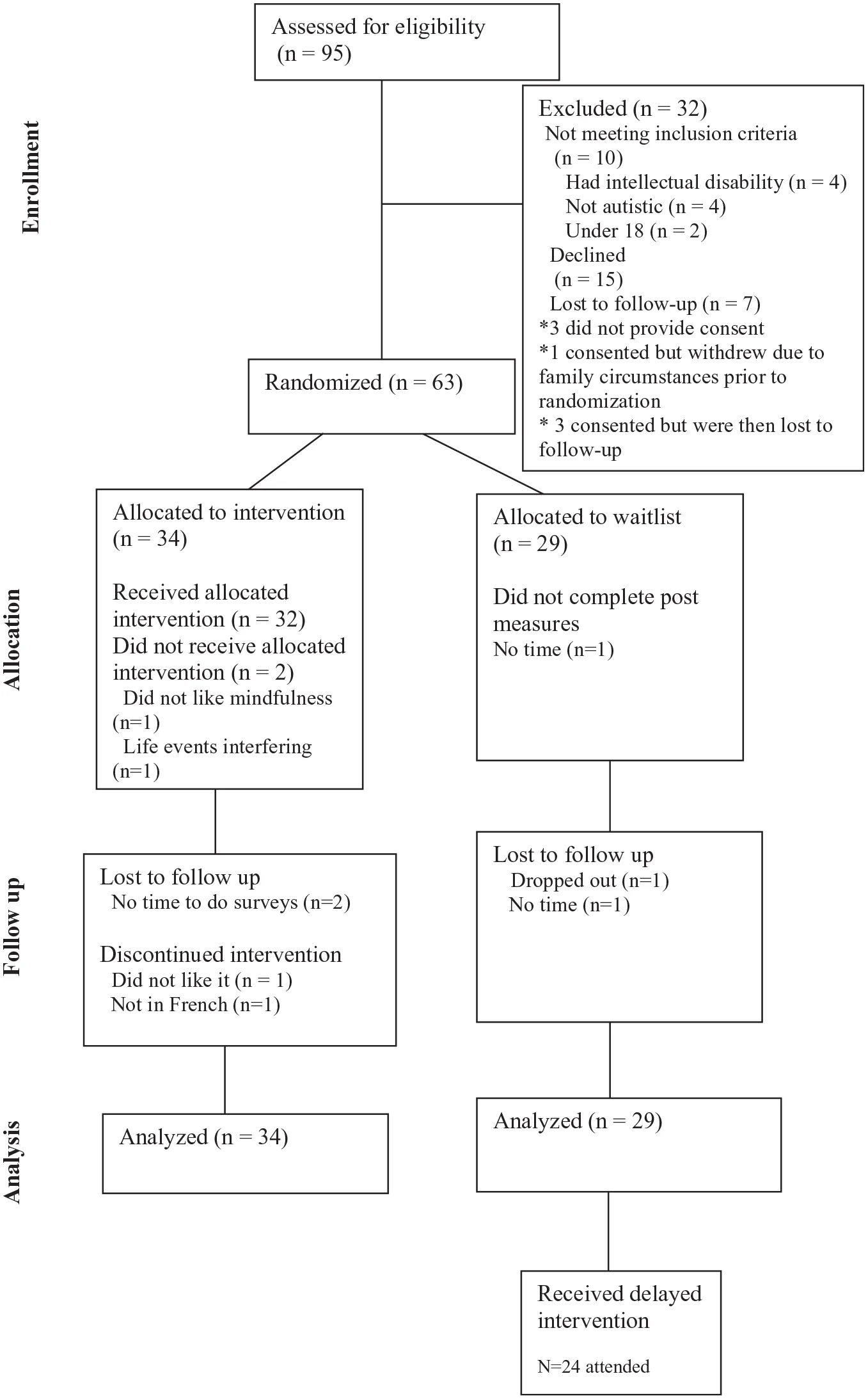
CONSORT diagram.

### Primary outcome

As indicated in Table 3, there was a significant group × time interaction for overall distress (DASS-21), with a medium effect size. Post hoc within-subject changes indicated that the mindfulness group did not show a significant reduction in overall distress from baseline to post-intervention (Week 7; (estimated marginal) mean difference = 3.49, standard error = 1.52, p = 0.14), and no change from Week 7 to follow-up (Week 15; mean difference = 2.53, standard error = 1.57, p = 0.66). There was, however, a significant change from pre-intervention to follow-up (difference = 6.02, standard error = 1.56, p = 0.0012). Similar changes were not observed in the WL arm (all *p*’s > 0.45). This pattern was also apparent for the DASS depression and stress subscale, but not for anxiety. Similar patterns were also seen in the secondary analysis (see Supplemental Table).

**Table 3.** Primary and secondary outcomes for mindfulness treatment (MI) and waitlist control (WL).

Measure	Group	Baseline M (SD) (*N*, MI = 34, WL = 29)	Week 7 M (SD) (*N*, MI = 30, WL = 27)	Week 15 M (SD) (N, MI = 28, WL = 26)	Group × time interaction effect (type III analysis of variance table with Satterthwaite’s method for linear mixed models) ANOVA eta^2^
DASS total	Treatment WL	26.74 (12.96) 25.28 (11.46)	22.30 (11.84) 28.26 (10.09)	18.93 (11.82) 24.65 (10.62)	*F* (2, 107.8) = 4.70, p = 0.01 **η** **=** **0.08**
Depression	Treatment WL	8.47 (4.26) 8.97 (4.36)	6.97 (4.00) 9.59 (4.72)	5.93 (4.08) 8.85 (5.23)	*F* (2, 108.8) = 3.11, p = 0.048 **η** **=** **0.05**
Anxiety	Treatment WL	7.06 (4.94) 6.72 (4.28)	6.00 (4.52) 7.44 (4.34)	5.43 (4.43) 5.77 (4.24)	*F* (2, 108.6) = 1.27, p = 0.28 **η** **=** **0.02**
Stress	Treatment WL	11.21 (5.00) 9.59 (4.25)	9.33 (4.53) 11.12 (3.73)	7.57 (4.48) 10.04 (3.64)	*F* (2, 106.7) = 7.32, p = 0.001 **η** **=** **0.12**
Warwick–Edinburgh Mental Wellbeing Scale (short-form)	Treatment WL	20.88 (4.36) 21.03 (3.96)	23.10 (4.56) 20.00 (4.53)	23.50 (4.11) 20.69 (4.04)	*F* (2, 111.9) = 6.93, p = 0.001 **η** **=** **0.11**
Self-Compassion Scale (short-form)	Treatment WL (*N* = 28)	29.91 (7.78) 30.11 (8.77)	36.23 (8.15) 29.85 (8.81)	38.89 (7.22) 31.19 (8.86)	*F* (2, 107.6) = 9.11, p = 0.00 **η** **=** **0.14**
Autistic Community Connectedness	Treatment WL	40.09 (8.93) 39.41 (14.01)	39.60 (9.64) 40.33 (13.06)	38.82 (10.55) 39.12 (13.60)	*F* (2, 108.8) = 0.06, p = 0.94 **η** **=** **0.00**
Interoception Sensory Questionnaire (8-item version)	Treatment WL	23.06 (8.83) 23.21 (8.91)	22.83 (8.84) 22.93 (7.92)	22.14 (8.84) 21.38 (8.42)	*F* (2, 107.9) = 0.00, p = 1.00 **η** **=** **0.00**
Five-Facet Mindfulness Questionnaire (short-form)	Treatment WL	69.12 (11.36) 70.79 (11.56)	74.37 (11.58) 70.78 (10.89)	78.00 (12.63) 71.96 (10.37)	*F* (2, 110.3) = 5.83, p = 0.003 **η** **=** **0.10**

### Secondary outcomes

As shown in Table 3, there were also significant group × time interactions for self-compassion, mindfulness, and mental wellbeing, each with medium or large effect sizes. Post hoc simple comparisons indicated that the mindfulness group showed a significant improvement in self-compassion from baseline to post-intervention (Week 7; mean difference = −5.43, standard error = 1.02, p < 0.001), but no significant change from Week 7 to follow-up (Week 15; mean difference = −2.34, standard error = 1.05, p = 0.167). There was also a significant change from pre-intervention to follow-up (mean difference = −7.77, standard error = 1.04). There were no significant changes for the WL group (all p’s > 0.69). This same pattern was observed for overall mindfulness (pre-intervention to Week 7; mean difference = −5.06, standard error = 1.48, p = 0.005; and pre-intervention to Week 15, mean difference = −8.04, standard error = 1.52, p < 0.001) and for mental wellbeing (pre-intervention to Week 7; mean difference = −2.12, standard error = 0.63, p = 0.006; pre-intervention to Week 15, mean difference = −2.40, standard error = 0.65, p = 0.002). Changes were not observed in the WL arm (all p > 0.32). Similar patterns emerged in the pooled analyses (see Supplemental Table).

Table 4 summarizes satisfaction ratings of 52 participants who completed the MI from both the treatment and WL conditions. The majority, 82.7%, indicated that the group content was relevant to them, that it gave them skills they can use in everyday life, that they plan to use these skills in the future, and that they felt supported and valued throughout the group. The majority of participants reported that the virtual nature of the program was convenient (86.7%) and only 15.4% reported that they would have preferred an in-person program (see Table 4). Although no checklist of side effects or harms related to the MI was administered, no adverse events were reported in the open text section of the satisfaction questionnaire, and no one withdrew from the treatment group because of reports of adverse events.

**Table 4.** Participants (*N* = 52) who agreed or strongly agreed with statements about virtual mindfulness intervention.

Item	*N* (%)
Satisfaction with program content	
It was easy to understand the content presented in my group	47 (90.1%)
The group content was relevant for me	43 (82.7%)
I thought the group was interesting	42 (80.8%)
The skills I have learned in this group have positively impacted social connections outside the group	19 (36.6%)
The project addressed goals that were important to me	37 (71.2%)
The group gave me skills that I can use in my everyday life	43 (82.7%)
I plan to continue to use the skills I learned in the future	43 (82.7%)
I would like to stay connected with the other participants	24 (46.2%)
I felt supported and valued throughout the group	43 (82.7%)
Satisfaction with technology/virtual aspects of program	
The web-conferencing software was easy to use	40 (76.9%)
I did not encounter many technological issues while trying to participate in the session	34 (65.4%)
I was able to find a quiet environment that allowed me to participate virtually in sessions	39 (75.0%)
I felt connected to the others in this online group, and I thought there was a strong sense of community, even though the sessions were virtual	24 (46.2%)
The online sessions were convenient and easier to attend than in-person sessions, as the online component removed potential barriers to participation, such as transportation requirements	45 (86.6%)
My comfort level with web-based technology has improved over the course of the sessions	20 (38.5%)
I would have preferred to be part of an in-person group	8 (15.4%)

### Discussion

This study provides additional support for a virtual brief synchronous mindfulness for autistic adults. A replication of changes was observed in this study as in prior research from our team, including significant changes in the mindfulness group compared to the WL control group. Consistent with the initial feasibility study, medium effect size changes were observed in overall distress and stress, self-compassion, and mindfulness. In addition, improvements in positive wellbeing were reported. No changes were observed in autistic community connectedness or interoceptive awareness, however. This study lends further support to the more general finding that mindfulness-based interventions can be helpful for some autistic people, and there are different ways in which such interventions can be delivered. For at least some individuals, virtual participation in a group synchronous format is beneficial. This study also suggests that the treatment dosage may not need to be very intensive to see change in mindfulness-related and clinical outcomes and for these changes to be maintained once programs end.

The current study found improvements in both self-compassion and mindfulness. Not all interventions with autistic people have detected changes in mindfulness, even when distress is reduced (Agius et al., 2023: Beck et al., 2020). Why this is the case is worth exploring. To our knowledge, this is the only study to be delivered in a virtual, group synchronous format. Both the Beck and Agius studies were also group-based, but they were held in person, prior to the pandemic and were standard MBSR interventions with slight modifications. In contrast, the current mindfulness program was quite different: There were fewer sessions in total, session length was shorter, all exercises were shorter, and the order of what was taught varied from standard MBSR. Finally, the current intervention included an autistic advisor co-facilitating, which has not been part of other interventions. Another explanation for a lack of differences in the prior studies relates to how mindfulness was measured, as opposed to how it was delivered. Both the Beck and Agius studies used a briefer mindfulness measure (the MAAS), which may be less sensitive to change than the FFMQ-SF when completed by autistic people. Further research on the most appropriate measures of mindfulness for autistic people is warranted.

Contrary to our hypothesis, no changes were observed in relation to autistic community connectedness. The autistic community connectedness measure was included in the current study because it was developed with and for autistic people and because of the qualitative observation in our prior research about the importance of connecting with other people through an autistic mindfulness program. One explanation for the lack of change may be related to the measure itself. Items focus on aspects of advocacy and connections built through active engagement with autistic people, neither of which were targets of the virtual MI. Many people did not talk about themselves at all within session, or their experiences as autistic people in their communities, rather focusing more specifically on learning mindfulness practices. There may also be a difference between feeling accepted and building social connections. In the satisfaction questionnaire, the majority of participants reported feeling supported and valued, whereas less than half wanted to stay connected to other participants after the group, and about one-third reported improved social connections outside of the group. It would be interesting to explore how different types of mental health interventions impact a sense of connectedness with autistic people. In addition, it would be valuable to explore other measures of autistic identity, and sense of connection with other people (autistic or more generally) as they relate to group-based mental health interventions.

Although changes were observed in both mindfulness and self-compassion, no changes were observed in interoception. It may be that a more refined or intensive intervention focusing on bodily sensations would have a stronger impact, and that such an intervention would be most relevant to people who struggle with interoceptive sensitivity. For example, aligning dimensions of interoceptive experience (ADIE), an interoception-focused intervention for autistic adults, has demonstrated reductions in anxiety for autistic adults (Quadt et al., 2021). Given the difficulties that some autistic people report attending to interoception, it would be important to explore how to support people with this more intensive internal focus in mindfulness-based programs and consider whether mindfulness would be an appropriate intervention for them. Some autistic people may find mindfulness practices focused on internal sensations (e.g. body scan) to be challenging and find other interventions like ADIE more acceptable. To our knowledge, although the interception measure used in the current study has been validated for this population, no studies have explored how different types of interventions may change sensitivity to internal sensations in autistic people using this measure. It has been suggested that more extensive measures focused on different aspects of interoception, along with physiological measures would be valuable (Loureiro et al., 2024).

This study is one of a few recent studies that are autistic-informed, group-based, and virtual in nature. Many studies have spoken about the challenges autistic people have accessing mental health services (e.g. Benevides et al., 2020; Dückert et al., 2023), as well as problems more generally in terms of the demands of in-person interventions, particularly in settings that have not been informed with an autistic lens. The current study illustrates that some of the stresses of in-person settings can be avoided through virtual interventions co-designed and delivered with autistic people, which may improve accessibility and reduce costs. Most participants found the virtual nature of the program to be convenient and reported that they would not have preferred something in person. The virtual nature of the program also allowed individuals who needed to move or take breaks, or make sounds during formal practices to do so without impacting the other participants (by muting themselves or turning off their camera) which cannot be done in person. It is interesting that changes were observed in this study, which only offered a total of 6 hours of intervention, compared to other mindfulness programs which can be much longer (e.g. 2-hour sessions, more weeks, with a silent retreat). This raises interesting questions about treatment dose, such as how much training is required to lead to change, how much home-based practice is necessary, and how important is sustained practice to change. It is possible that simply understanding more about what mindfulness is, and learning a few simple strategies that may be helpful that are autistic-informed, may be enough to lead to change for at least some individuals. The unique benefits of being in a group setting with other autistic people as opposed to learning mindfulness in an asynchronous way would be valuable to explore in future research.

### Limitations and next steps

A limitation in our study is that we did not meet our recruitment goal, and therefore may have been underpowered to detect certain differences between groups, although many differences would remain non-significant with our planned sample size assuming similar effect sizes. Second, although this study advanced our prior work by adding a WL control group, it would have been stronger to adopt an active treatment comparison. Research needs to focus on how this synchronous virtual intervention compares to other virtual mental health interventions (e.g. cognitive behavioral therapy (CBT), autistic peer support), as well as systematically comparing different mindfulness treatment modalities (in person to virtual; asynchronous to synchronous). These kinds of more rigorous active designs may also help answer important questions about which interventions are the best fit for which participants: Are there some people who would be more interested in one type of group than another and would certain people report greater benefits in one type of group than another? Furthermore, more in-depth study, combining quantitative and qualitative data, is required to understand which aspects of mindfulness-based intervention are most helpful, and why some individuals improve more than others. Future research should also assess harms from the MI in a more systematic way than was done in the current study, and should also include measures of fidelity, and neuro-affirming adaptations to more standard mindfulness-based techniques. Continued research into when mindfulness-based interventions would be most helpful is needed. Finally, this study included individuals both with and without mood and anxiety disorder diagnoses. Further refinement is needed to ascertain the role of mindfulness-based interventions as a mental health treatment in addition to it being a potential tool to promote mental health in those with subclinical levels of distress, prior to seeking mental health services.

## Supplemental Material

sj-docx-1-aut-10.1177_13623613251340101 – Supplemental material for Virtual group–based mindfulness for autistic adults: A randomized controlled trial Supplemental material, sj-docx-1-aut-10.1177_13623613251340101 for Virtual group–based mindfulness for autistic adults: A randomized controlled trial by Yona Lunsky, Madelaine Carter, Sue Hutton, Jan Wozniak, Sheng Chen and Jonathan Weiss in Autism

sj-pdf-2-aut-10.1177_13623613251340101 – Supplemental material for Virtual group–based mindfulness for autistic adults: A randomized controlled trial Supplemental material, sj-pdf-2-aut-10.1177_13623613251340101 for Virtual group–based mindfulness for autistic adults: A randomized controlled trial by Yona Lunsky, Madelaine Carter, Sue Hutton, Jan Wozniak, Sheng Chen and Jonathan Weiss in Autism

## References

[bibr1-13623613251340101] Agius H. Luoto A.-K. Backman A. Eriksdotter C. Jayaram-Lindström N. Bölte S. Hirvikoski T. (2023). Mindfulness-based stress reduction for autistic adults: A feasibility study in an outpatient context. Autism, 28(2), 403–414. 10.1177/13623613231172809 PMC10851647 37190953

[bibr2-13623613251340101] Ali D. O’Brien S. Hull L. Kenny L. Mandy W. (2023). ‘The key to this is not so much the technology. It’s the individual who is using the technology’: Perspectives on telehealth delivery for autistic adults during the COVID-19 pandemic. Autism, 27(2), 552–564. 10.1177/13623613221108010 PMC10076158 35791666

[bibr3-13623613251340101] Baer R. A. Smith G. T. Hopkins J. Krietemeyer J. Toney L. (2006). Using self-report assessment methods to explore facets of mindfulness. Assessment, 13(1), 27–45. 10.1177/1073191105283504 16443717

[bibr4-13623613251340101] Beck K. B. Greco C. M. Terhorst L. A. Skidmore E. R. Kulzer J. L. McCue M. P. (2020). Mindfulness-based stress reduction for adults with autism spectrum disorder: Feasibility and estimated effects. Mindfulness, 11(5), 1286–1297. 10.1007/s12671-020-01345-2

[bibr5-13623613251340101] Benevides T. W. Shore S. M. Palmer K. Duncan P. Plank A. Andresen M.-L. Caplan R. Cook B. Gassner D. Hector B. L. Morgan L. Nebeker L. Purkis Y. Rankowski B. Wittig K. Coughlin S. S. (2020). Listening to the autistic voice: Mental health priorities to guide research and practice in autism from a stakeholder-driven project. Autism, 24(4), 822–833. 10.1177/1362361320908410 PMC7787673 32429818

[bibr6-13623613251340101] Bonete S. Molinero C. Ruisanchez D. (2023). Emotional dysfunction and interoceptive challenges in adults with autism spectrum disorders. Behavioral Sciences, 13(4), 312. 10.3390/bs13040312 PMC10136046 37102826

[bibr7-13623613251340101] Bornemann B. Herbert B. M. Mehling W. E. Singer T. (2015). Differential changes in self-reported aspects of interoceptive awareness through 3 months of contemplative training. Frontiers in Psychology, 5, 1504. 10.3389/fpsyg.2014.01504 PMC4284997 25610410

[bibr8-13623613251340101] Botha M. (2020). Autistic community connectedness as a buffer against the effects of minority stress [Doctoral dissertation, University of Surrey, Guildford]. 10.15126/THESIS.00854098

[bibr9-13623613251340101] Botha M. Dibb B. Frost D. M. (2022). ‘It’s being a part of a grand tradition, a grand counter-culture which involves communities’: A qualitative investigation of autistic community connectedness. Autism, 26(8), 2151–2164. 10.1177/13623613221080248 PMC9597163 35318862

[bibr10-13623613251340101] Braden B. B. Pagni B. A. Monahan L. Walsh M. J. M. Dixon M. V. Delaney S. Ballard L. Ware J. E. (2022). Quality of life in adults with autism spectrum disorder: Influence of age, sex, and a controlled, randomized mindfulness-based stress reduction pilot intervention. Quality of Life Research, 31(5), 1427–1440. 10.1007/s11136-021-03013-x PMC9012783 34655389

[bibr11-13623613251340101] Brede J. Cage E. Trott J. Palmer L. Smith A. Serpell L. Mandy W. Russell A. (2022). “We have to try to find a way, a clinical bridge”—autistic adults’ experience of accessing and receiving support for mental health difficulties: A systematic review and thematic meta-synthesis. Clinical Psychology Review, 93, 102131. 10.1016/j.cpr.2022.102131 35180632

[bibr12-13623613251340101] Cage E. Cranney R. Botha M. (2022). Brief report: Does autistic community connectedness moderate the relationship between masking and wellbeing? Autism in Adulthood, 4(3), 247–253. 10.1089/aut.2021.0096 PMC9645674 36606159

[bibr13-13623613251340101] Cai R. Y. Edwards C. Love A. M. Brown L. Gibbs V. (2024). Self-compassion improves emotion regulation and mental health outcomes: A pilot study of an online self-compassion program for autistic adults. Autism, 28, 2572–2585. 10.1177/13623613241235061 38584374

[bibr14-13623613251340101] Cohen J. (1992). A power primer. Psychological Bulletin, 112(1), 155–159. 10.1037/0033-2909.112.1.155 19565683

[bibr15-13623613251340101] Conner C. M. White S. W. (2018). Brief report: Feasibility and preliminary efficacy of individual mindfulness therapy for adults with autism spectrum disorder. Journal of Autism and Developmental Disorders, 48(1), 290–300. 10.1007/s10803-017-3312-0 28921306

[bibr16-13623613251340101] Curnow E. Rutherford M. Maciver D. Johnston L. Prior S. Boilson M. Shah P. Jenkins N. Meff T. (2023). Mental health in autistic adults: A rapid review of prevalence of psychiatric disorders and umbrella review of the effectiveness of interventions within a neurodiversity informed perspective. PLOS ONE, 18(7), Article e0288275. 10.1371/journal.pone.0288275 PMC10343158 37440543

[bibr17-13623613251340101] Dückert S. Gewohn P. König H. Schöttle D. Konnopka A. Rahlff P. Erik F. Vogeley K. Schulz H. David N. Peth J. (2023). Barriers and needs in mental healthcare of adults with autism spectrum disorder in Germany: A qualitative study in autistic adults, relatives, and healthcare providers. BMC Psychiatry, 23(1), Article 528. 10.1186/s12888-023-05026-x PMC10362719 37479974

[bibr18-13623613251340101] Edwards C. Gibbs V. Love A. M. A. Brown L. Cai R. Y. (2024). A qualitative exploration of an autism-specific self-compassion program: The ASPAA. Autism, 28(6), 1419–1430. 10.1177/13623613241234097 PMC11134985 38383994

[bibr19-13623613251340101] Fiene L. Ireland M. J. Brownlow C. (2018). The Interoception Sensory Questionnaire (ISQ): A scale to measure interoceptive challenges in adults. Journal of Autism and Developmental Disorders, 48(10), 3354–3366. 10.1007/s10803-018-3600-3 29748924

[bibr20-13623613251340101] Gaigg S. B. Flaxman P. E. McLaven G. Shah R. Bowler D. M. Meyer B. Roestorf A. Haenschel C. Rodgers J. South M. (2020). Self-guided mindfulness and cognitive behavioural practices reduce anxiety in autistic adults: A pilot 8-month waitlist-controlled trial of widely available online tools. Autism, 24(4), 867–883. 10.1177/1362361320909184 PMC7418273 32267168

[bibr21-13623613251340101] Garfinkel S. N. Tiley C. O’Keeffe S. Harrison N. A. Seth A. K. Critchley H. D. (2016). Discrepancies between dimensions of interoception in autism: Implications for emotion and anxiety. Biological Psychology, 114, 117–126. 10.1016/j.biopsycho.2015.12.003 26724504

[bibr22-13623613251340101] Harris L. Gilmore D. Hanks C. Coury D. Moffatt-Bruce S. Garvin J. H. Hand B. N. (2022). “It was surprisingly equivalent to the appointment I had in person”: Advantages and disadvantages of synchronous telehealth for delivering primary care for autistic adults. Autism, 26(6), 1573–1580. 10.1177/13623613211060589 PMC9149139 34847744

[bibr23-13623613251340101] Hartley M. Dorstyn D. Due C. (2019). Mindfulness for children and adults with autism spectrum disorder and their caregivers: A meta-analysis. Journal of Autism and Developmental Disorders, 49(10), 4306–4319. 10.1007/s10803-019-04145-3 31342444

[bibr24-13623613251340101] Henry J. D. Crawford J. R. (2005). The short-form version of the Depression Anxiety Stress Scales (DASS-21): Construct validity and normative data in a large non-clinical sample. British Journal of Clinical Psychology, 44(2), 227–239. 10.1348/014466505X29657 16004657

[bibr25-13623613251340101] Kabat-Zinn J. (2013). Full catastrophe living: Using the wisdom of your body and mind to face stress, pain, and illness (Revised and updated edition). Bantam Books.

[bibr26-13623613251340101] Kiep M. Spek A. A. Hoeben L. (2015). Mindfulness-based therapy in adults with an autism spectrum disorder: Do treatment effects last? Mindfulness, 6(3), 637–644. 10.1007/s12671-014-0299-x

[bibr27-13623613251340101] Lai M.-C. Kassee C. Besney R. Bonato S. Hull L. Mandy W. Szatmari P. Ameis S. H. (2019). Prevalence of co-occurring mental health diagnoses in the autism population: A systematic review and meta-analysis. The Lancet Psychiatry, 6(10), 819–829. 10.1016/S2215-0366(19)30289-5 31447415

[bibr28-13623613251340101] Loureiro F. Ringold S. Aziz-Zadeh L. (2024). Interoception in autism: A narrative review of behavioral and neurobiological data. Psychology Research and Behavior Management, 17, 1841–1853. 10.2147/PRBM.S410605 PMC11075678 38716258

[bibr29-13623613251340101] Lunsky Y. Redquest B. Albaum C. Hutton S. Share M. Share-Strom D. Weiss J. (2022). Virtual group–based mindfulness intervention for autistic adults: A feasibility study. Mindfulness, 13(7), 1706–1718. 10.1007/s12671-022-01909-4 PMC9189269 35729967

[bibr30-13623613251340101] Maitland C. A. Rhodes S. O’Hare A. Stewart M. E. (2021). Social identities and mental well-being in autistic adults. Autism: The International Journal of Research and Practice, 25(6), 1771–1783. 10.1177/13623613211004328 PMC8323333 34011188

[bibr31-13623613251340101] Mazurek M. O. Pappagianopoulos J. Brunt S. Sadikova E. Nevill R. Menezes M. Harkins C. (2023). A mixed methods study of autistic adults’ mental health therapy experiences. Clinical Psychology & Psychotherapy, 30(4), 767–779. 10.1002/cpp.2835 PMC10372197 36708045

[bibr32-13623613251340101] Neff K. D. (2003). The development and validation of a scale to measure self-compassion. Self and Identity, 2(3), 223–250. 10.1080/15298860309027

[bibr33-13623613251340101] Pellicano E. Heyworth M. (2023). The foundations of autistic flourishing. Current Psychiatry Reports, 25(9), 419–427. 10.1007/s11920-023-01441-9 PMC10506917 37552401

[bibr34-13623613251340101] Quadt L. Garfinkel S. N. Mulcahy J. S. Larsson D. E. O. Silva M. Jones A.-M. Strauss C. Critchley H. D. (2021). Interoceptive training to target anxiety in autistic adults (ADIE): A single-center, superirotiy randomized controlled trial. EClinical Medicine, 39(2021), 101042. 10.1016/j.eclinm.2021.101042 PMC8350004 34401684

[bibr35-13623613251340101] Raes F. Pommier E. Neff K. D. Van Gucht D. (2011). Construction and factorial validation of a short form of the Self-Compassion Scale. Clinical Psychology & Psychotherapy, 18(3), 250–255. 10.1002/cpp.702 21584907

[bibr36-13623613251340101] Sizoo B. B. Kuiper E. (2017). Cognitive behavioural therapy and mindfulness based stress reduction may be equally effective in reducing anxiety and depression in adults with autism spectrum disorders. Research in Developmental Disabilities, 64, 47–55. 10.1016/j.ridd.2017.03.004 28342404

[bibr37-13623613251340101] Spek A. A. Van Ham N. C. Nyklícek I. (2013). Mindfulness-based therapy in adults with an autism spectrum disorder: A randomized controlled trial. Research in Developmental Disabilities, 34(1), 246–253. 10.1016/j.ridd.2012.08.009 22964266

[bibr38-13623613251340101] Stecher C. Pagni B. A. Cloonan S. Vink S. Hill E. Ogbeama D. Delaney S. Braden B. B. (2023). App-based meditation habits maintain reductions in depression symptoms among autistic adults. Autism, 28(6), 1487–1502. 10.1177/13623613231200679 37837362

[bibr39-13623613251340101] Stewart-Brown S. Tennant A. Tennant R. Platt S. Parkinson J. Weich S. (2009). Internal construct validity of the Warwick-Edinburgh Mental Well-being Scale (WEMWBS): A Rasch analysis using data from the Scottish Health Education Population Survey. Health and Quality of Life Outcomes, 7(1), 15. 10.1186/1477-7525-7-15 PMC2669062 19228398

[bibr40-13623613251340101] Suzman E. Williams Z. J. Feldman J. I. Failla M. Cascio C. J. Wallace M. T. Niarchou M. Sutcliffe J. S. Wodka E. Woynaroski T. G. (2021). Psychometric validation and refinement of the Interoception Sensory Questionnaire (ISQ) in adolescents and adults on the autism spectrum. Molecular Autism, 12(1), Article 42. 10.1186/s13229-021-00440-y PMC8185943 34099040

[bibr41-13623613251340101] Westerberg B. Holländare F. Bejerot S. (2023). An internet-based behavioral intervention for adults with autism spectrum disorder – A randomized controlled trial and feasibility study. Internet Interventions, 34, 100672. 10.1016/j.invent.2023.100672 PMC10523266 37772160

