# Supplementary material for: Virtual group–based mindfulness for autistic adults: A randomized controlled trial
Source: Autism. 2025 Jul 1;29(10):2489–500. doi: 10.1177/13623613251340101 (PMC13687638; doi:10.1177/13623613251340101)
Supplement: Supplementary file 1 [file sj-pdf-2-aut-10.1177_13623613251340101.pdf]

camh | Azrieli Adult Neurodevelopmental Centre

Research Study

Virtual Mindfulness Groups: Do they help with stress in autistic adults?

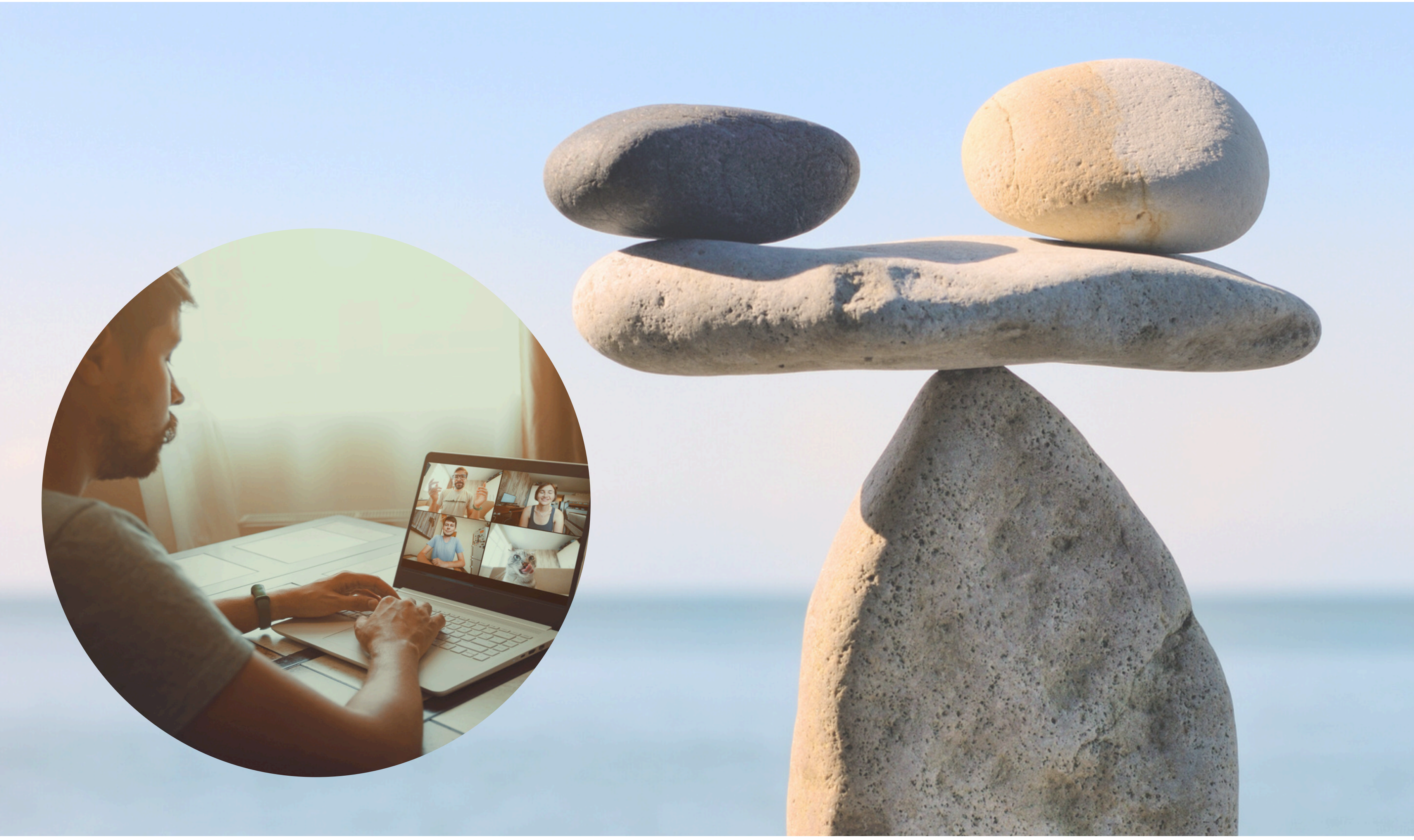

What is mindfulness?

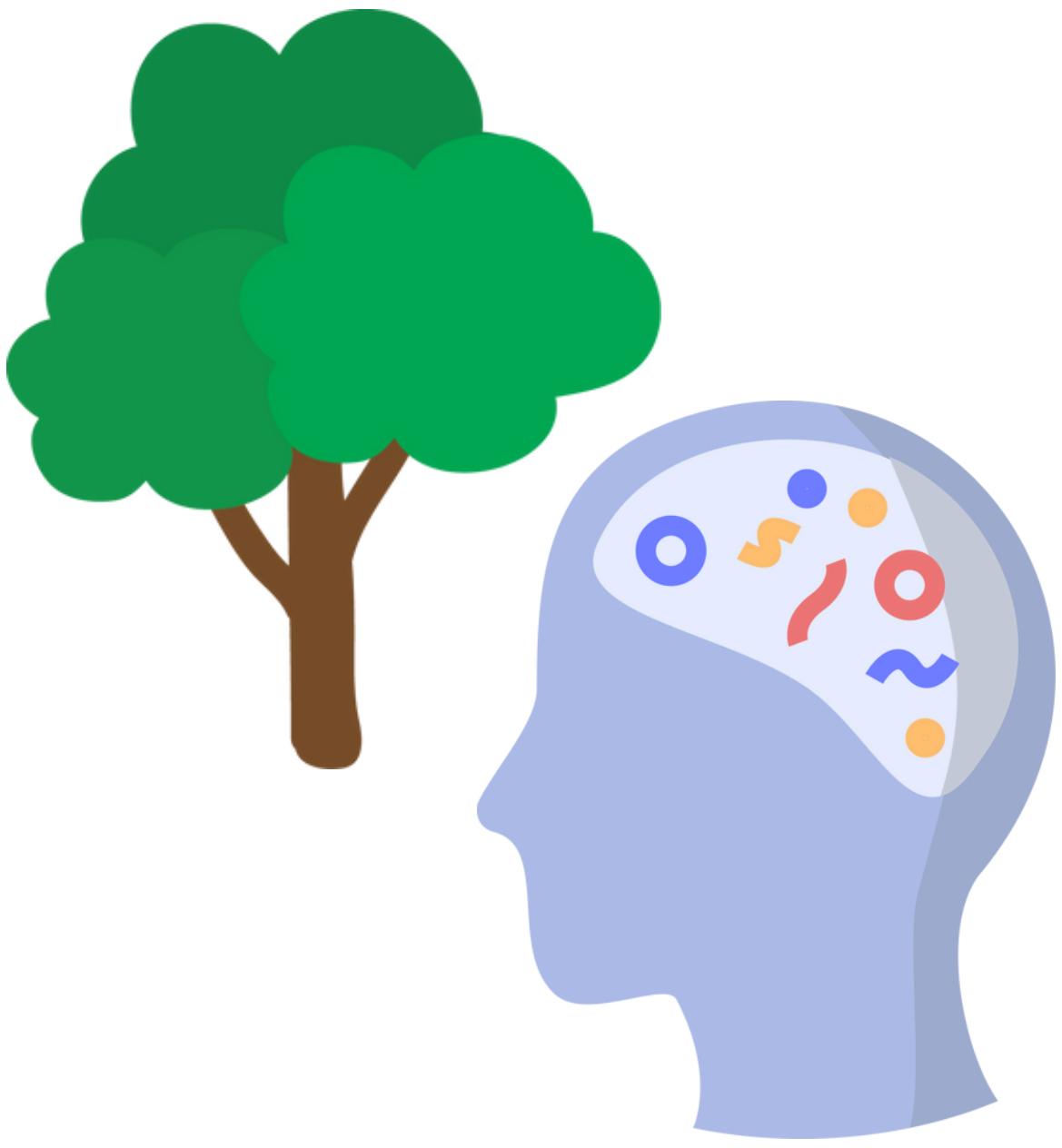

Mindfulness is **paying attention** to your **thoughts, feelings, and what is happening around you** right now.

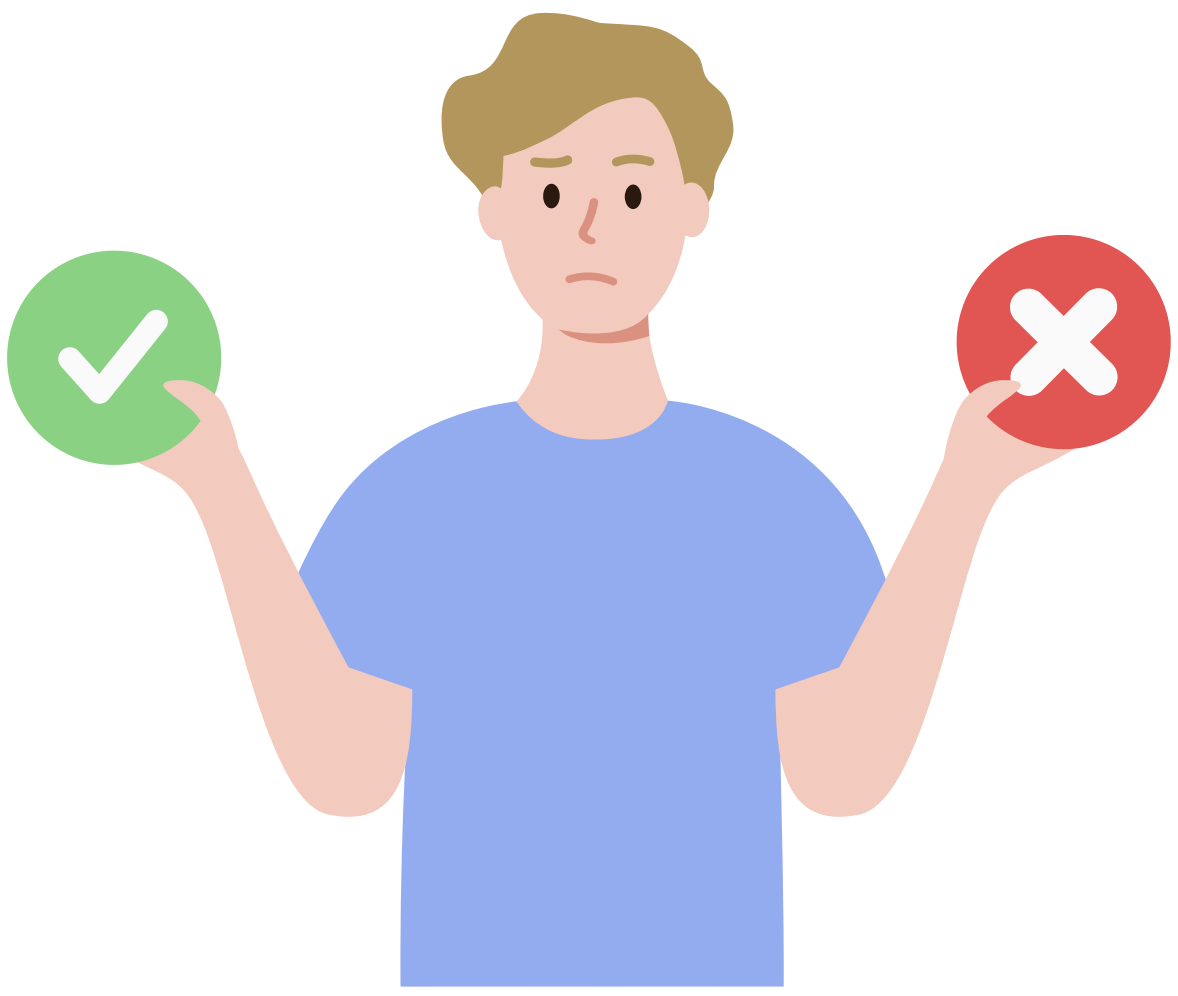

When you do mindfulness, you try to notice these things **without labeling them as good or bad.**

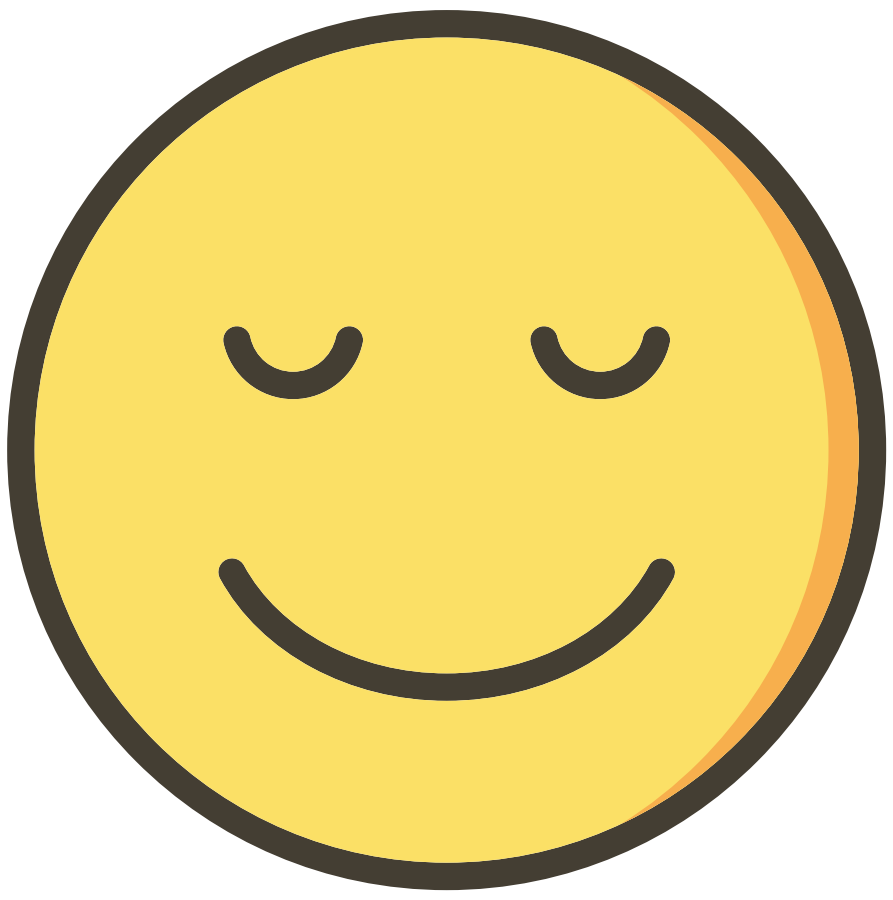

Mindfulness can help some people to feel **calm and less stressed.**

Why did we do this research?

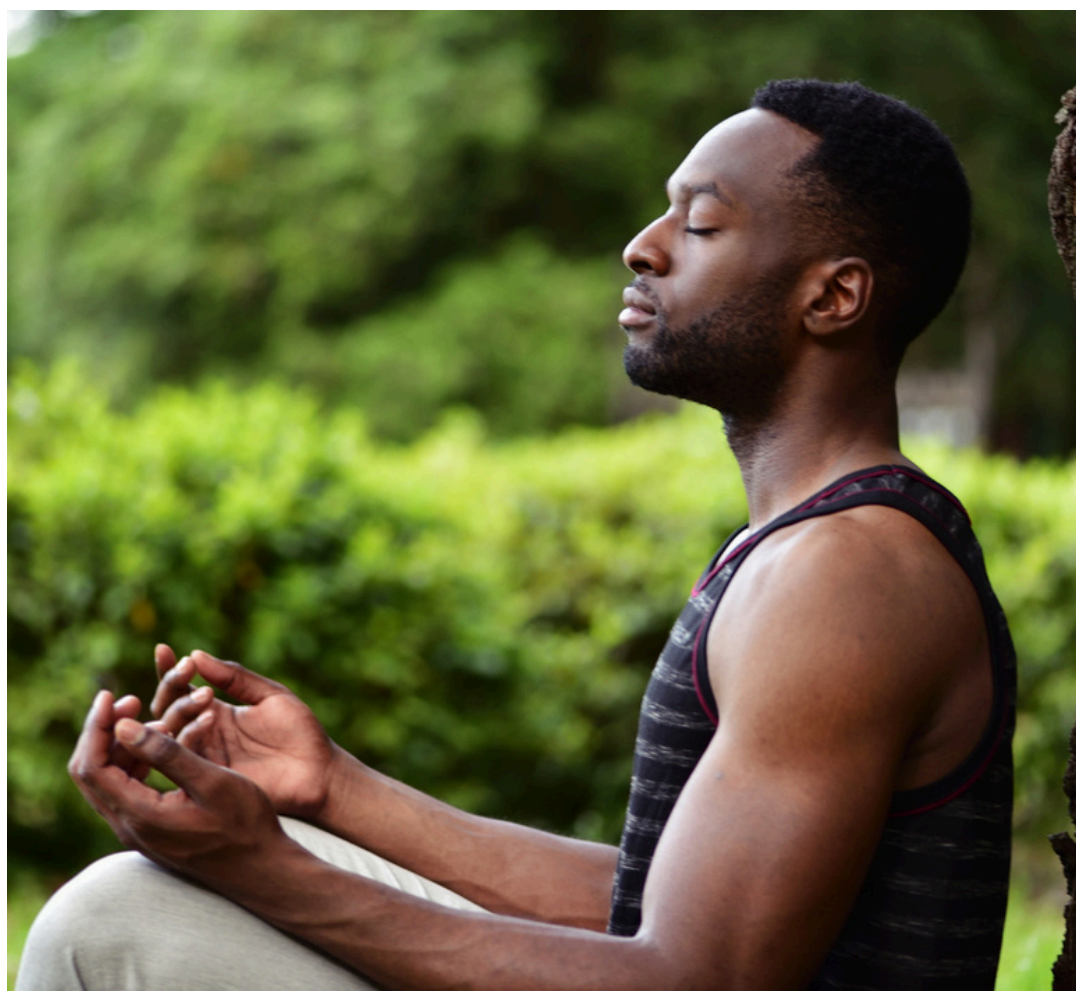

Some studies have shown that learning mindfulness helps autistic people to feel **less stressed.**

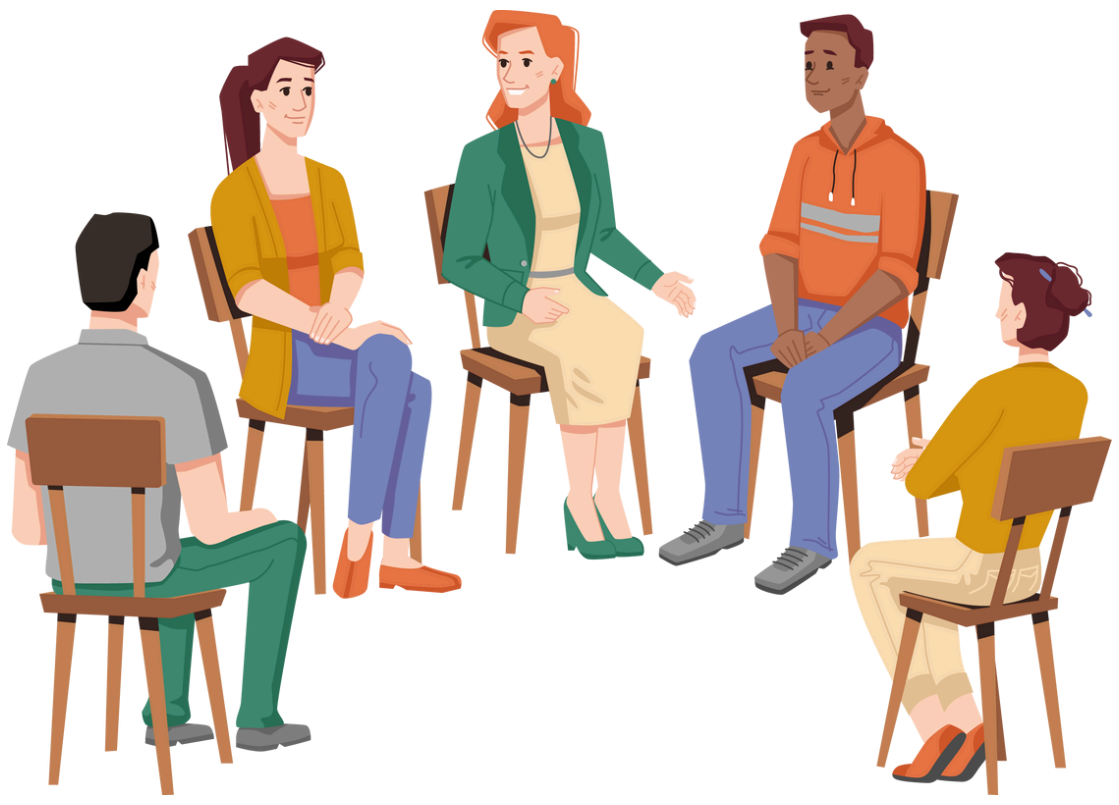

But most of these studies were done with **people sitting in a room together.**

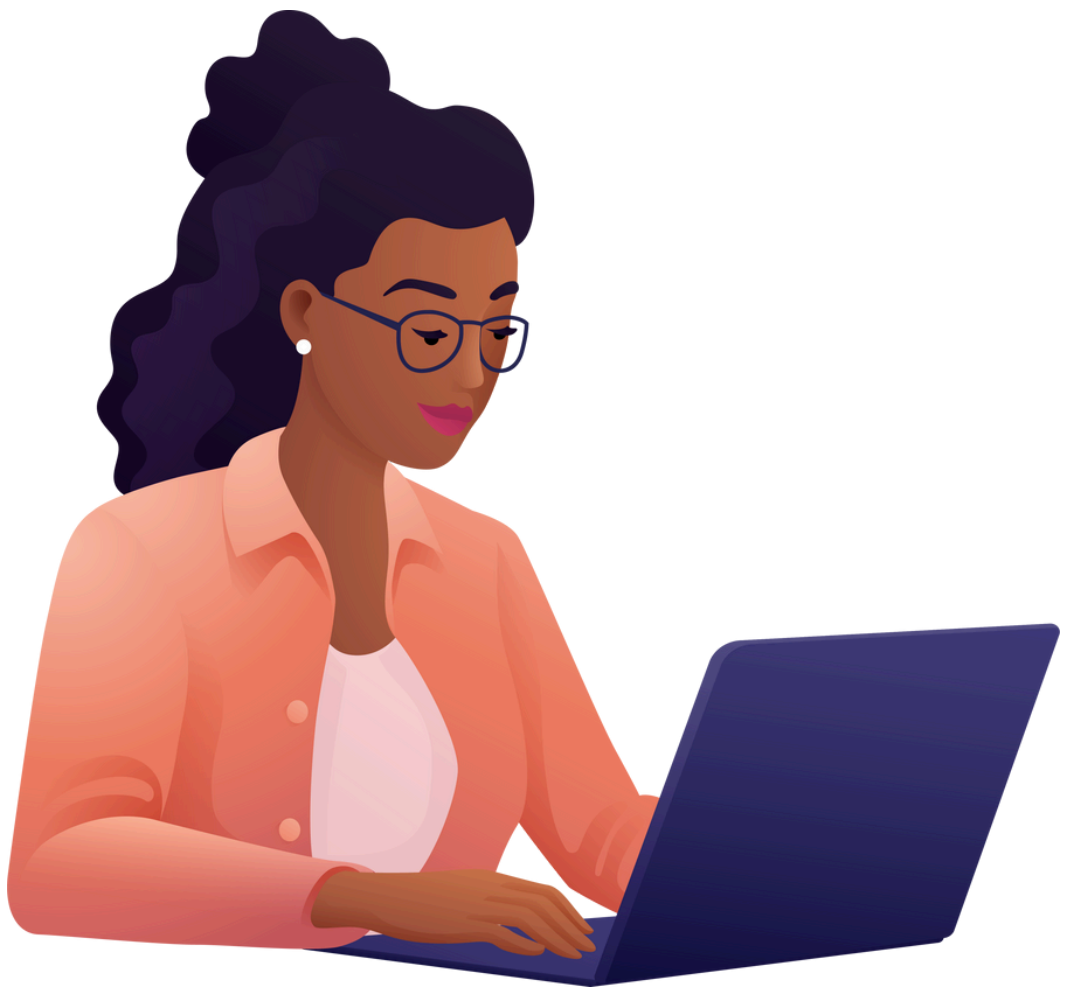

We wanted to know if learning **mindfulness online** in a group would help improve autistic adults' **mental health.**

What did we do?

We made an online group mindfulness course for Autistic adults.

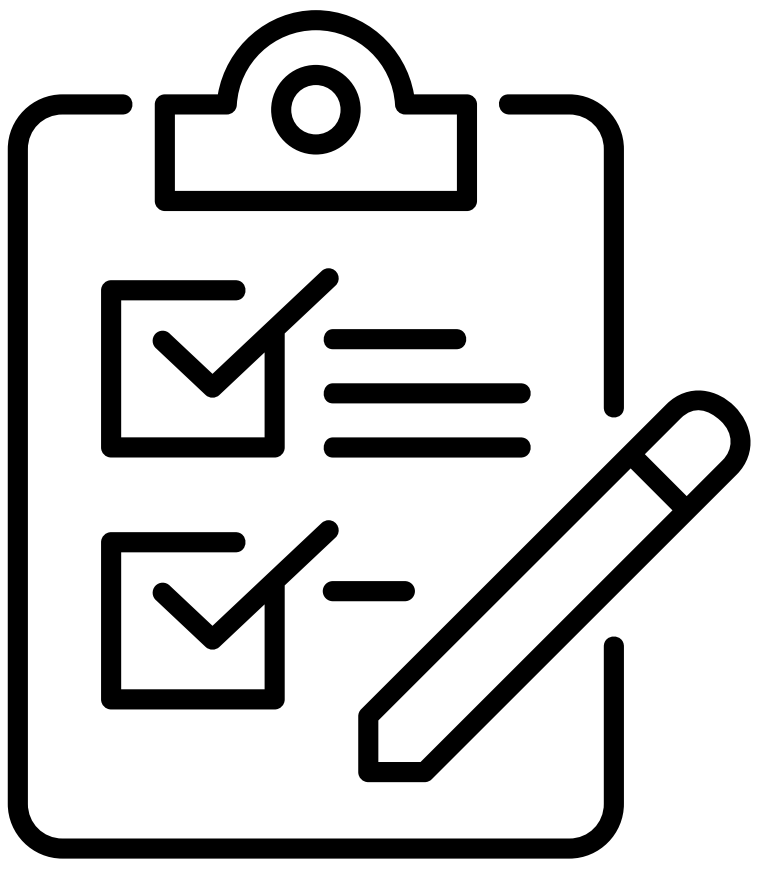

After agreeing to be in our project people filled out surveys about their **mental health.**

Then we randomly put 63 people into **two groups:**

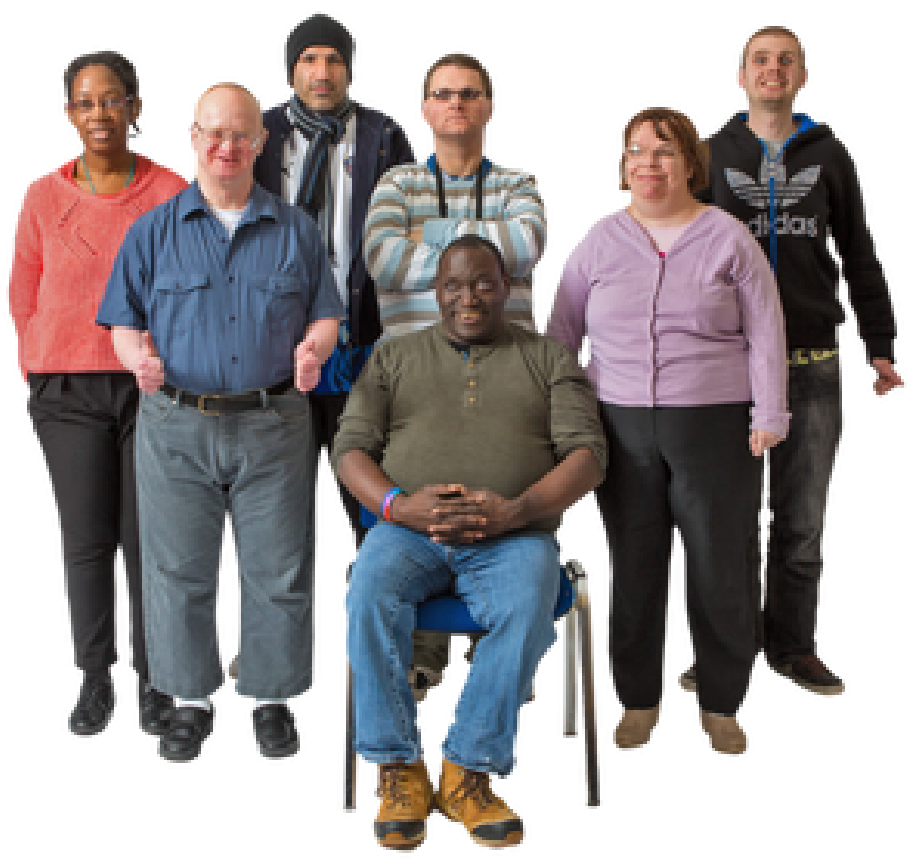

Mindfulness

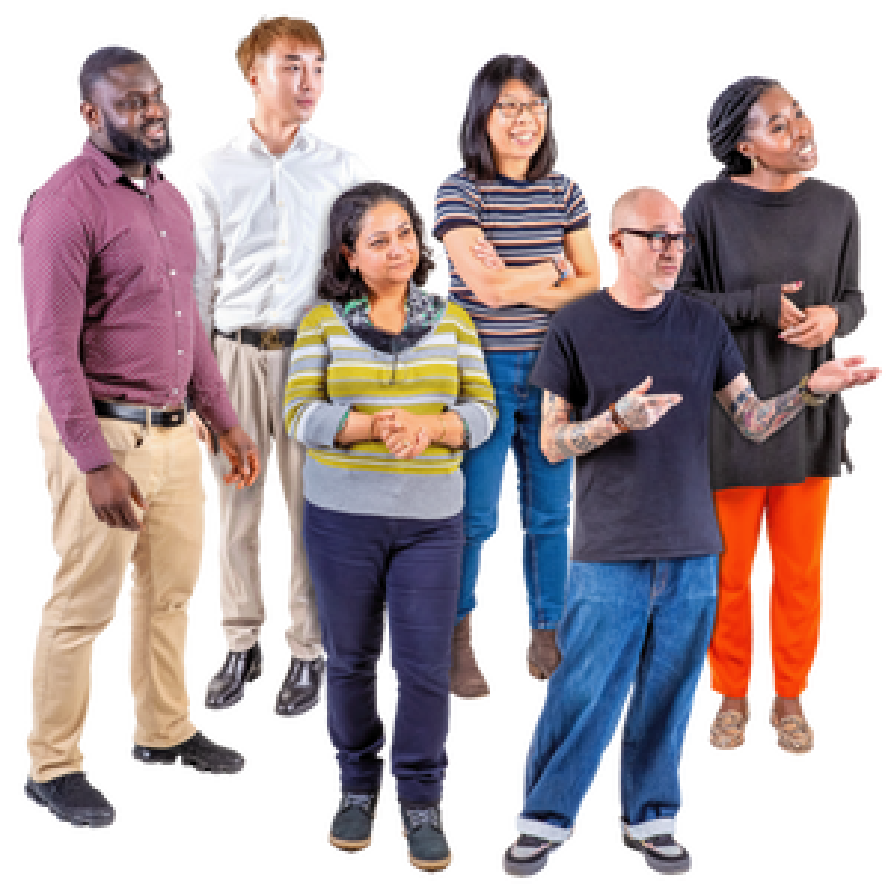

No mindfulness

(The **no mindfulness** group got to do mindfulness after the project was finished.)

What did we do?

We studied how people who did mindfulness and people who did not do mindfulness felt.

Before the course started, both groups filled out surveys about how they were feeling.

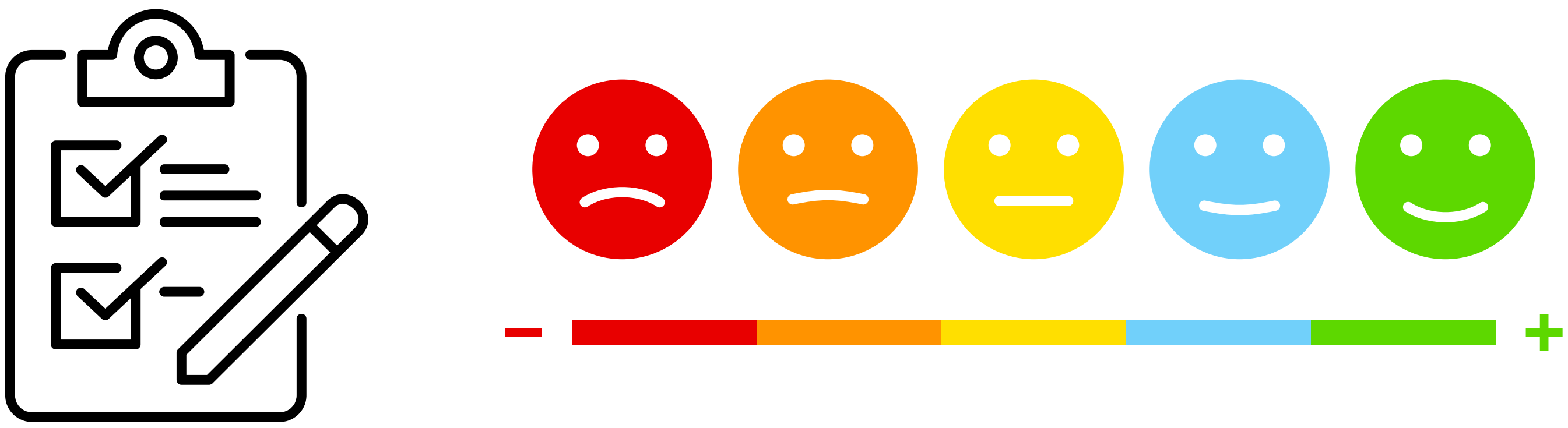

Both groups filled out surveys **again** about how they were feeling **7 and 15 weeks later.**

We wanted to know **how people's feelings changed** over time **in the** two groups **(mindfulness** and **no mindfulness) .**

What did we learn?

Surveys from people who learned mindfulness showed:

- they felt **less stressed**
- they were **more mindful**
- they had **better wellbeing**
- they had **more compassion or kindness for themselves**

These **changes were still there** 8 weeks later.

Surveys from people in the 'no mindfulness' group showed they **did not feel any differently during this time.**

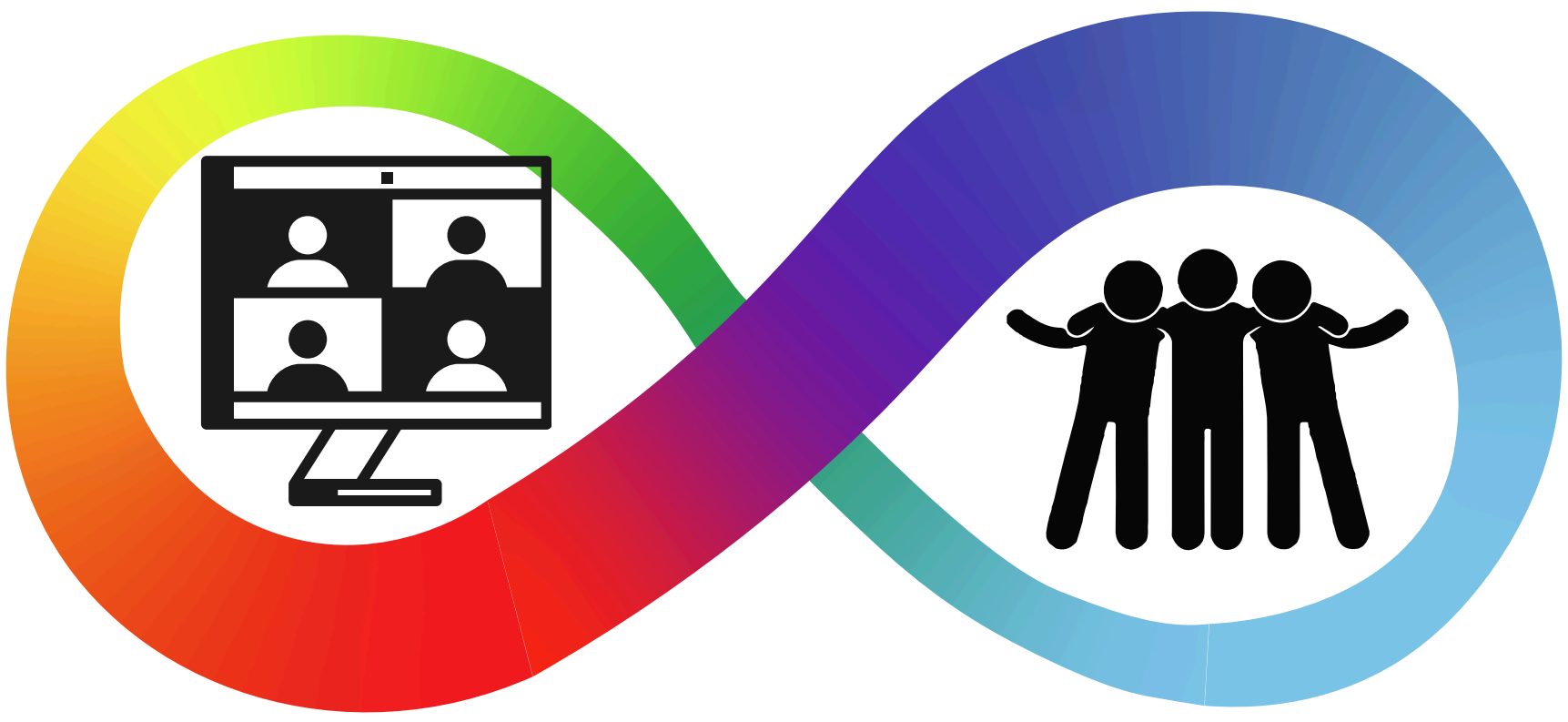

What does this mean?

This tells us that **we can teach groups of autistic people to do mindfulness** online together.

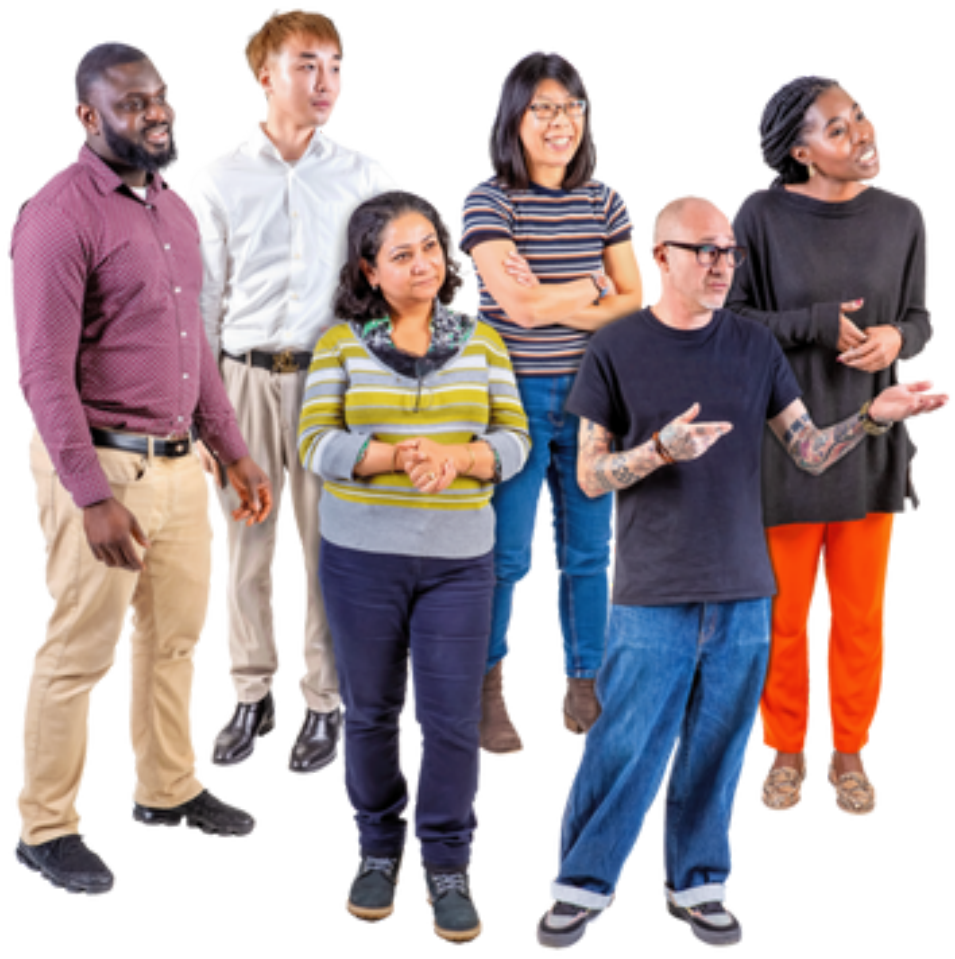

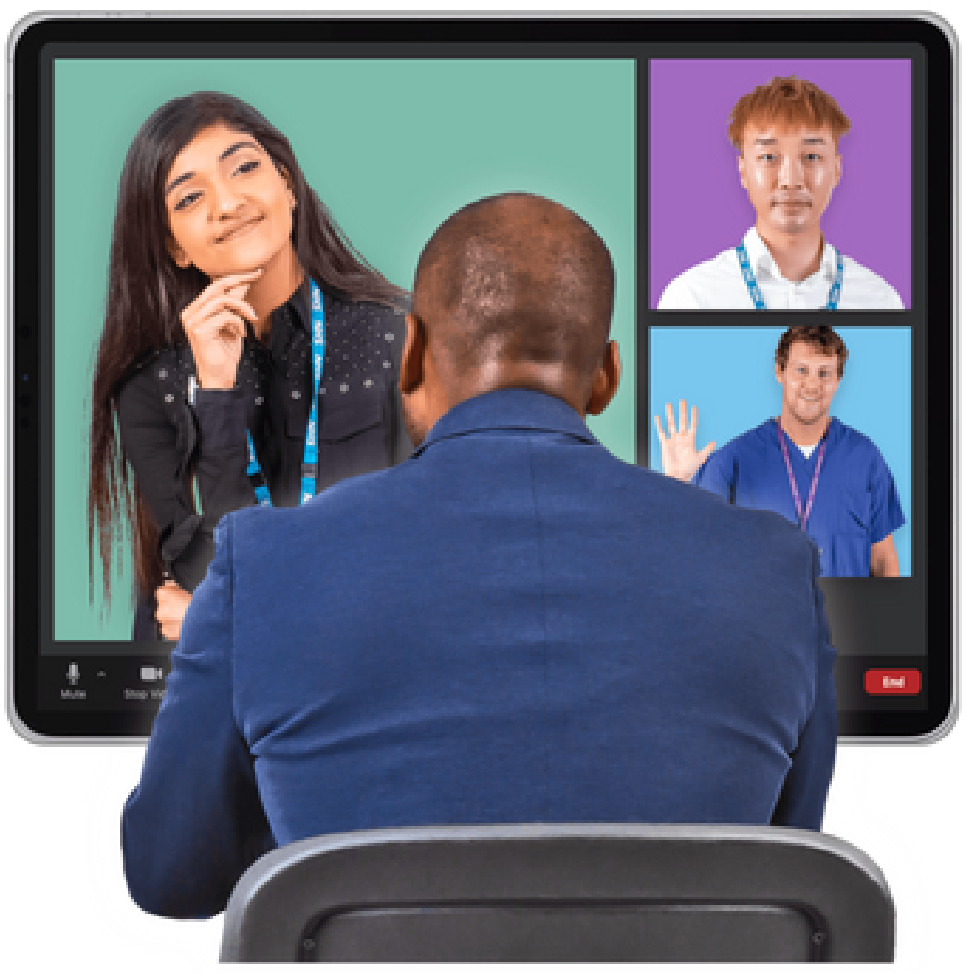

This can help some autistic people with their **mental health.**

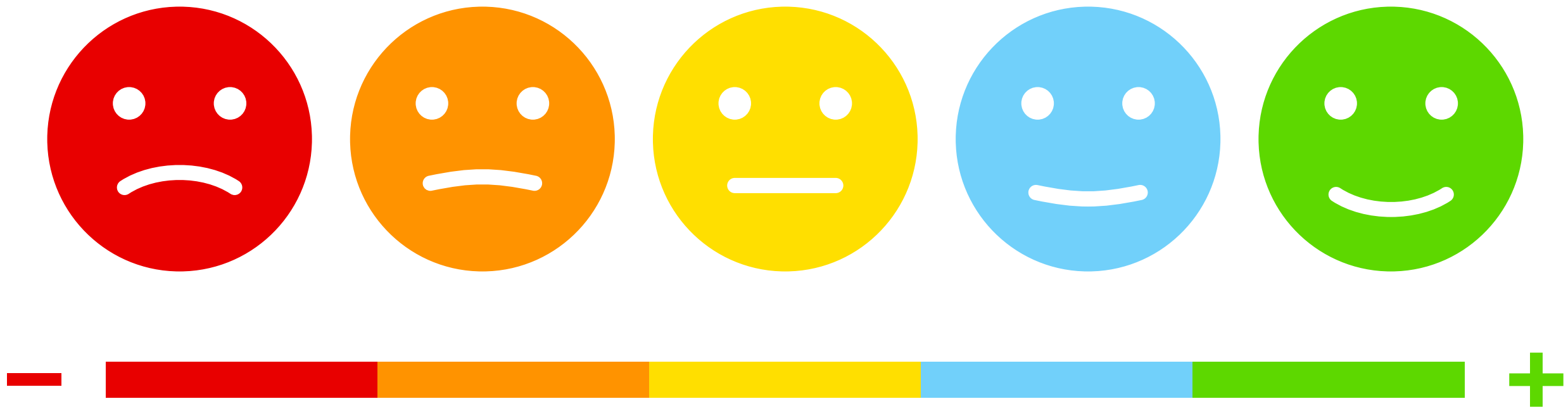

What does this mean?

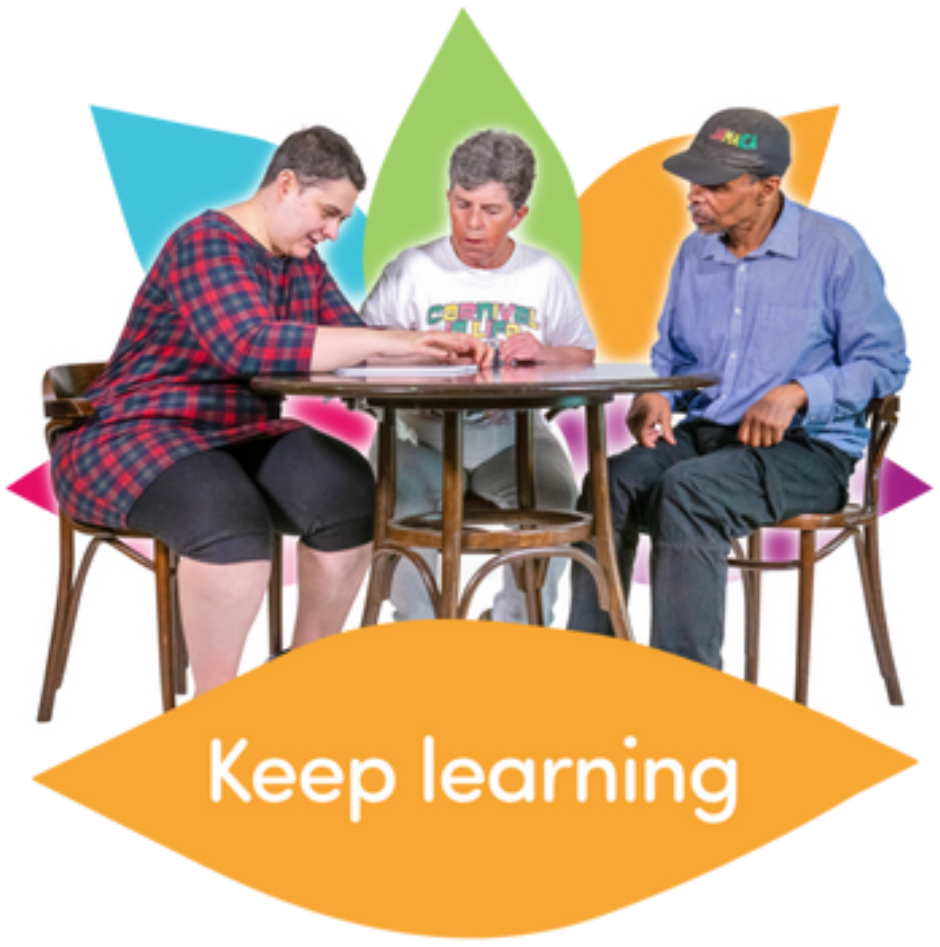

We need to do more research to find out how much **mindfulness training** people need.

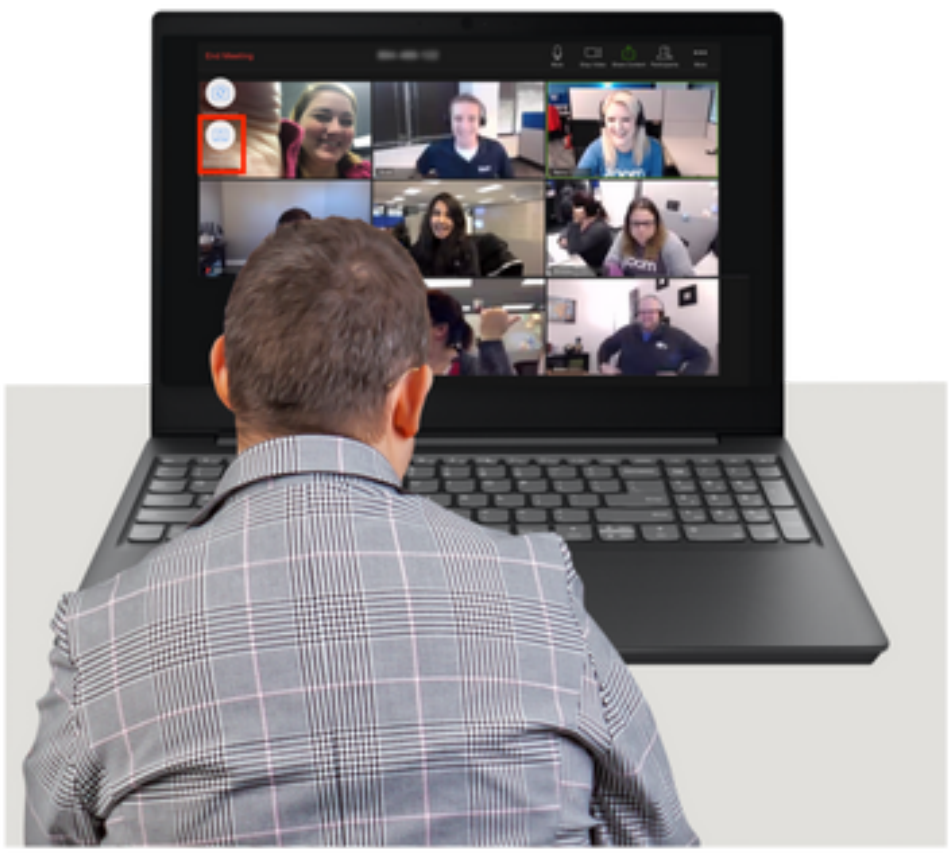

We need to find out if it is better to learn mindfulness **in person or online.**

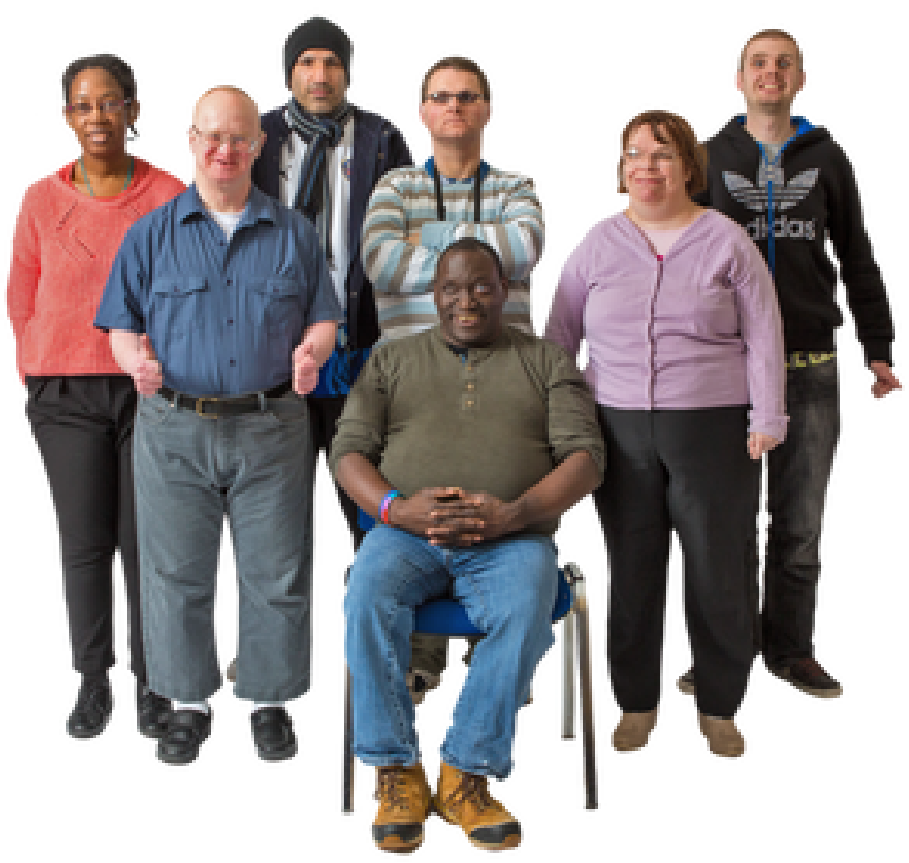

We need to find out if it is better to learn **as part of a group or alone.**

Title: Virtual group based mindfulness for autistic adults: A randomised controlled trial

Authors: *Yona Lunsky, Madelaine Carter, Sue Hutton, Jan Wozniak, Sheng Chen, Jonathan Weiss*

*This research study is posted in a journal called **Autism**.*

This study was funded by The Public Health Agency of Canada and the Azrieli Foundation.

This summary was created by staff at the Azrieli Adult Neurodevelopmental Centre at CAMH.

We thank the self-advocate advisors who worked together with our team to create this research summary.

camh | Azrieli Adult Neurodevelopmental Centre

HCARDD Health Care Access Research and Developmental Disabilities

made with photosymbols®
