# Supplementary material for: Virtual group–based mindfulness for autistic adults: A randomized controlled trial
Source: Autism. 2025 Jul 1;29(10):2489–500. doi: 10.1177/13623613251340101 (PMC13687638; doi:10.1177/13623613251340101)
Supplement: Supplementary file 2 [file sj-docx-1-aut-10.1177_13623613251340101.docx]

**Supplementary Table**

| Measure | Group | Baseline  M (SD)  (N,  Treatment=34,  WL=29) | Week 7  M (SD)  (N,  Treatment=50,  WL=27) | Week 15  M (SD)  (N,  Treatment=46,  WL=26) | Group by time interaction effect (Type III Analysis of Variance Table with Satterthwaite's method for linear mixed models)  ANOVA eta^2^ |
| --- | --- | --- | --- | --- | --- |
| DASS total  Depression  Anxiety  Stress | Treatment  WL  Treatment  WL  Treatment  WL  Treatment  WL | 26.74 (12.96)  25.28 (11.46)  8.47 (4.26)  8.97 (4.36)  7.06 (4.94)  6.72 (4.28)  11.21 (5.00)  9.59 (4.25) | 21.54 (11.70)  28.26 (10.09)  7.58 (5.03)  9.59 (4.72)  5.78 (4.43)  7.44 (4.34)  9.10 (5.79)  11.12 (3.73) | 19.80 (11.50)  24.65 (10.62)  5.65 (3.78)  8.85 (5.23)  6.22 (4.68)  5.77 (4.24)  7.93 (4.28)  10.04 (3.64) | F (2, 148.0)=4.14, p =.018  **ᶯ =0.05**  F (2, 150.6)=2.63, p=0.075  **ᶯ =0.03**  F (2, 149.4)=2.57, p=0.08  **ᶯ =0.03**  F (2, 148.7)=5.16, p=0.007  **ᶯ =0.06** |
| Warwick–Edinburgh Mental Wellbeing Scale  (short-form) | Treatment  WL | 20.88 (4.36)  21.03 (3.96) | 22.54 (6.47)  20.00 (4.53) ^#^ | 23.28 (4.11)  20.69 (4.04) | F (2, 153.9)=3.52, p=0.03  **ᶯ =0.04** |
| Self Compassion Scale  (short-form) | Treatment  WL (N=28) | 29.91 (7.78)  30.11 (8.77) | 35.74 (9.32)  29.85 (8.81) | 37.98 (8.81)  31.19 (8.86) | F (2, 149.2)=6.77, p=0.001  **ᶯ =0.08** |
| Autistic Community Connectedness | Treatment  WL | 40.09 (8.93)  39.41 (14.01) | 39.34 (13.49)  40.33 (13.06) | 40.61 (11.97)  39.12 (13.60) | F(2, 152.8)=0.63, p=0.53  **ᶯ =0.00** |
| Interoception Sensory Questionnaire (8-item version) | Treatment  WL | 23.06 (8.83)  23.21 (8.91) | 23.50 (11.11)  22.93 (7.92) | 20.89 (8.51)  21.38 (8.42) | F (2, 149.0)=0.16, p=0.85  **ᶯ =0.00** |
| Five-Facet Mindfulness Questionnaire (short-form) | Treatment  WL | 69.12 (11.36)  70.79 (11.56) | 72.16 (18.91)  70.78 (10.89) | 76.89 (12.84)  71.96 (10.37) | F (2, 153.2)=2.04, p=0.13  **ᶯ =0.03** |

*Primary and Secondary Outcomes for Treatment and Waitlist Control Group **

*The measures of the WL following treatment were pooled with the Treatment group at Week7 and Week 15.
